# Particle analysis of surgical lung biopsies from deployed and non-deployed US service members during the Global War on Terrorism

**DOI:** 10.1371/journal.pone.0301868

**Published:** 2024-04-11

**Authors:** Leslie Hayden, James M. Lightner, Stacy Strausborger, Teri J. Franks, Nora L. Watson, Michael R. Lewin-Smith

**Affiliations:** 1 Institute for Functional Materials and Devices, Lehigh University, Bethlehem, Pennsylvania, United States of America; 2 Environmental Microscopy Laboratory, Joint Pathology Center, Silver Spring, Maryland, United States of America; 3 Pulmonary and Mediastinal Pathology, Joint Pathology Center, Silver Spring, Maryland, United States of America; 4 Department of Research Programs, Walter Reed National Military Medical Center, Bethesda, Maryland, United States of America; 5 Environmental Pathology, Joint Pathology Center, Silver Spring, Maryland, United States of America; West Virginia University School of Medicine, UNITED STATES

## Abstract

The role that inhaled particulate matter plays in the development of post-deployment lung disease among US service members deployed to Southwest Asia during the Global War on Terrorism has been difficult to define. There is a persistent gap in data addressing the relationship between relatively short-term (months to a few years) exposures to high levels of particulate matter during deployment and the subsequent development of adverse pulmonary outcomes. Surgical lung biopsies from deployed service members and veterans (DSMs) and non-deployed service members and veterans (NDSMs) who develop lung diseases can be analyzed to potentially identify residual deployment-specific particles and develop associations with pulmonary pathological diagnoses. We examined 52 surgical lung biopsies from 25 DSMs and 27 NDSMs using field emission scanning electron microscopy (FE-SEM) with energy dispersive x-ray spectroscopy (EDS) to identify any between-group differences in the number and composition of retained inorganic particles, then compared the particle analysis results with the original histopathologic diagnoses. We recorded a higher number of total particles in biopsies from DSMs than from NDSMs, and this difference was mainly accounted for by geologic clays (illite, kaolinite), feldspars, quartz/silica, and titanium-rich silicate mixtures. Biopsies from DSMs deployed to other Southwest Asia regions (SWA-Other) had higher particle counts than those from DSMs primarily deployed to Iraq or Afghanistan, due mainly to illite. Distinct deployment-specific particles were not identified. Particles did not qualitatively associate with country of deployment. The individual diagnoses of the DSMs and NDSMs were not associated with elevated levels of total particles, metals, cerium oxide, or titanium dioxide particles. These results support the examination of particle-related lung disease in DSMs in the context of comparison groups, such as NDSMs, to assist in determining the strength of associations between specific pulmonary pathology diagnoses and deployment-specific inorganic particulate matter exposure.

## Introduction

During the Global War on Terrorism (GWOT), between September 2001 and August 2021, 3.7 million US service members were deployed. Many service members had multiple deployments, with the majority occurring before September 2011 [1, 2]. Following deployment, a proportion of these personnel developed adverse medical outcomes, including pulmonary pathology. The degree to which specific exposures contribute to or cause these adverse medical outcomes is difficult to quantify, as contemporary measurements of exposures are often lacking, and proxy data retrospectively estimating exposures may introduce errors. There are also difficulties in assessing the impact of multiple, often overlapping exposures that occur during deployment. Data collected for individual chemicals may not accurately reflect the potential harm of the entire deployed environment. In addition, pulmonary pathology is influenced by both environmental and genetic factors. Other exposures, notably inhalation of fumes from burn pits, industrial pollution, vehicle exhaust, blast injuries and embedded fragments are also of concern [3, 4]. Exposures to volatile organic compounds may be difficult or impossible to accurately measure in formalin-fixed lung tissue months or years after exposure. However, inorganic particles retained in lung specimens can be evaluated to assess this subset of exposures of concern.

Surgical lung biopsy specimens from deployed service members and veterans (DSMs) obtained for the clinically indicated work-up and diagnosis of severe pulmonary conditions provide high quality diagnostic data that can potentially support associations with prior environmental exposures. The interval between the end of deployment and time of biopsy may influence the results, as a proportion of particles are removed (cleared) and others are newly inhaled during this time. However, the results can be correlated with dates and durations of deployment, which, while not a measurement of explicit exposure, “embodies time spent in a diverse foreign environment containing potentially dangerous exposures” [5]. Garshick et al., 2019, observed that there is a lack of published data to assess the long-term (respiratory) effects of repeated particulate matter (PM) or other air pollution exposure during deployment, and specifically, to quantify chronic adverse pulmonary outcomes attributable to short durations (months to a few years) of high levels of exposure [6].

In 2021, we reported the findings of particle analysis in surgical lung biopsies from DSMs and non-deployed service members and veterans (NDSMs) using scanning electron microscopy with energy dispersive x-ray spectroscopy (SEM/EDS) [7]. At the time, this analysis was limited by both the equipment available and time resources to support active pulmonary consultations. Field emission scanning electron microscopy (FE-SEM) with energy dispersive x-ray spectroscopy (EDS) is a technique that enables higher resolution imaging and analysis of particles in situ. In 2022, we reported our method of in situ analysis of retained particles in surgical lung biopsy specimens using an automated FE-SEM/EDS technique [8].

In this study we used automated FE-SEM/EDS to characterize the morphology and chemical composition of retained inorganic particles found in surgical lung biopsies from DSMs and NDSMs with a variety of non-neoplastic pulmonary diagnoses. We included surgical lung biopsy specimens from 25 DSMs, the largest sample from a deployed cohort examined by FE-SEM/EDS reported to date, along with surgical lung biopsies obtained during the same period from 27 NDSMs.

In an ideal research environment, population values for inorganic particle loads corrected for site of biopsy, patient age, gender, and smoking history would be available for service members without overt lung disease. These could then be compared with quantitative measurements for DSMs, and to both DSMs and NDSMs with specific non-neoplastic pulmonary diagnoses. Because these data do not exist, we examined surgical lung biopsies from NDSMs as a comparison group.

We hypothesized that there are differences in the number and composition of retained inorganic particles in surgical lung biopsies from DSMs and NDSMs who received their diagnoses from the same expert pulmonary pathologists during the same time interval. Additionally, we addressed potential confounding factors in interpreting the particle analysis results: patient demographics, biopsy site, smoking status, military service characteristics, and time interval between end of deployment and biopsy.

To test our hypothesis, we compared the particle analysis results between surgical lung biopsy specimens from DSMs and NDSMs. Among DSMs we compared particle analysis results from the different deployment regions within Southwest Asia (SWA) and to NDSMs. For both DSMs and NDSMs we compared particle analysis results with the histopathological non-neoplastic pulmonary diagnoses rendered for the specimens. This analysis provides useful data to further assess the association between inhaled inorganic particulate matter and post-deployment pulmonary pathology.

## Materials and methods

### Specimen selection

Surgical lung biopsy specimens from 52 patients were included. All patients were US service members and veterans; 25 patients were previously deployed to Southwest Asia during the Global War on Terrorism (DSMs), and 27 were not deployed (NDSMs). All surgical lung biopsies were obtained during the same time interval and the biopsies were performed as part of clinical evaluations for the diagnosis of pulmonary pathologies. The patients had a variety of clinical presentations; those of the DSMs developed post deployment. The surgical lung biopsy specimens were selected retrospectively from among second opinion consultations performed by subspecialist pulmonary and mediastinal pathologists at the former Armed Forces Institute of Pathology (AFIP) and the Joint Pathology Center (JPC). Selection was predicated on adequate formalin-fixed paraffin-embedded surgical lung biopsy specimens being present in the JPC Tissue Repository. Specimens were retrieved from the Repository under a Walter Reed National Military Medical Center institutional review board (WRNMMC-IRB) approved research protocol (WRNMMC-2020-0317). Our project was granted a waiver of informed consent as defined in 32 CFR 219.116(f) by the WRNMMC-IRB.

#### Military service and deployment information

Military service and deployment information was obtained from the Defense Manpower Data Center (DMDC) under an IRB-approved research protocol. Many DSMs had multiple deployments of various lengths. The assignment of deployment region was based on the region where the DSM served the longest: Iraq, Afghanistan, and SWA-Other. The category SWA-Other includes Kuwait, Qatar, Bahrain, and Kyrgyzstan. The duration of deployment was rounded to months.

#### Smoking

A smoker/non-smoker designation was based on the two study pathologists’ (TJF and MLS) review of the surgical pathology reports for smoking-related pathological terms, such as smoking related diffuse parenchymal lung disease (SRDPLD) and respiratory bronchiolitis, when smoking history was not provided at the time of original consultation. A histology-based approach to assess smoking status when it has not been provided in other records has been used by other authors [9].

#### Site and time of biopsy

The site and time of biopsy were obtained from former AFIP and JPC records. Biopsies from one lobe were examined in 30 cases, two lobes in 14 cases, and three lobes in eight cases. The left lung was sampled less often than the right (Table 1).

**Table 1:** Lung lobe sampling count by service member deployment status.

Deployment status	3 Lobes	2 Lobes	1 Lobe	Right	Left	Right/left not specified	Lobe not specified
**DSM**	3	6	16	18	7	1	4
**NDSM**	5	8	14	17	9	1	3
**Total**	8	14	30	35	16	2	7

The distribution of the biopsies and deployment end dates overlap; the median deployment end date was February 2009 and the median biopsy dates were February 2012 and October 2011 for the DSMs and NDSMs, respectively (S1 Fig).

### Comparative patient demographics

Patients’ demographics and military status are summarized in Table 2. The age differences between the DSMs and NDSMs at time of biopsy were insignificant (one-tail P-value, 14%). Army NDSMs were more likely to be smokers and had a higher mean age (40 versus 34 years) than Army DSMs. Air Force service members (DSMs and NDSMs) had a higher mean age than the total cohort. The only officers were Air Force DSMs. There were more female DSMs (5) than NDSMs (2), but otherwise the military service demographics were similar between DSMs and NDSMs.

**Table 2:** Service member demographics.

**Deployed Service Members (DSMs)**					
**Characteristic**	**Army**	**Air Force**	**Marine Corps**	**Navy**	**All Service**
**Count**	15	6	2	2	25
**Age at first deployment (Average, in years)**	30	36	30	30	32
**Age at biopsy (Average, in years)**	34	43	33	33	36
**Deployment length (Average, in months)**	12	8	12	6	11
**Months between end of last deployment and biopsy**	27	48	15	30	31
**Male**	12	5	1	2	20
**Female**	3	1	1	0	5
**Enlisted**	15	2	2	2	21
**Officer**	0	4	0	0	4
**Non-smoker**	9	5	2	1	17
**Smoker**	6	1	0	1	8
**Non-deployed service members (NDSMs)**					
**Characteristic**	**Army**	**Air Force**	**Marine Corps**	**Navy**	**All Service**
**Count**	14	6	2	5	27
**Age at biopsy (Average, in years)**	40	44	27	37	40
**Male**	12	6	2	5	25
**Female**	2	0	0	0	2
**Enlisted**	14	5	2	5	26*
**Officer**	0	0	0	0	0
**Non-smoker**	4	4	2	3	13
**Smoker**	10	2	0	2	14

*The rank of one NDSM was not known.

The mean ages of DSMs were greater than the mean ages of the total deployed US military population but were within one year for Army, the branch of service that represented 15 of 25 (60%) of the DSMs in our study [2]. There were five women in our deployed cohort (20%), which is greater than the proportion of women (10%) in the total deployed population [2].

### Particle analysis

The FE-SEM/EDS analysis was performed by scientists who were unaware of the patients’ deployment, demographic, smoking, and diagnostic information. For a detailed discussion of tissue preparation and the FE-SEM/EDS analytical method, please see Hayden et al. 2022 [8].

#### Specimen preparation

Five-micron-thick serial sections were cut from the formalin fixed, paraffin embedded (FFPE) tissue blocks at the JPC in Silver Spring, MD. A hematoxylin and eosin (H&E) stained slide was prepared and marked by a pathologist (MLS) to identify potential areas of accumulated inhaled PM. The adjacent section was mounted on a highly polished carbon planchet (Ted Pella, Inc. Redding, CA) for FE-SEM/EDS analysis. Carbon planchets were placed on a hot plate and warmed until the paraffin was absorbed by the planchet. Samples were then coated with approximately 20 to 25 nm of carbon in a high vacuum carbon evaporator (Denton, BenchTop Turbo, Moorestown, NJ) to prevent sample charging under the electron beam.

#### FE-SEM/EDS in-situ particle analysis

This study was conducted using a Hitachi SU8220 cold-field-emission (CFE) scanning electron microscope (Hitachi, Japan). For each sample approximately 300 fields were collected and analyzed to document a 1000 μm x 1000 μm sample area, regardless of the number or size of the regions of interest marked on the corresponding H&E slides. All fields were analyzed at a magnification of 2000x.

Automated particle analyses were performed using a Bruker XFlash 6|60 EDS (Bruker, Germany). Data collection was automated using the Feature Analysis and the Jobs applications found in the Bruker ESPRIT 2.3 software (Bruker, Germany). The analytical conditions used for analysis result in a detection limit of approximately 0.1 weight percent for most elements.

#### Chemistry and morphology

Following the successful completion of automated particle analysis, the spectrum of individual particles as determined by the Feature Analysis application were reviewed and the classification of each particle was confirmed or changed. Particles were then further classified into particle groups (e.g., clays, iron oxides, and feldspars). Using EDS data alone, in the absence of crystallographic data, it is not possible to determine whether a particle such as SiO_2_ is quartz (mineral) or silica glass (manufactured).

In many but not all samples, the particles existed in clusters or agglomerations with overlap between particles resulting in EDS spectra that are mixed signals of two or more particle types. Mixed spectra took two forms. In one form, the spectra were clearly recognizable mineral compositions with a trace metal inclusion: for example, titanium dioxide with a cerium peak. These spectra would be counted both as a titanium dioxide particle and a cerium particle. The second type of mixed spectra was far more common and was from true mixtures where the individual components cannot be identified due to agglomeration or clusters. These were characterized as “mixed,” and designated in this study as iron (Fe)-rich or titanium (Ti)-rich.

Despite the limitations of the technique, this method allowed for the efficient collection of a large particle dataset without introducing operator selection bias. The particle counts and area totals reported from these lung biopsy samples are lower limits, with the actual particle density being higher.

### Diagnoses

Between 2020 and 2022, we analyzed surgical lung biopsy specimens obtained from DSMs and NDSMs undergoing clinical evaluation for non-neoplastic diagnoses, referred for expert pulmonary pathology consultative review at the former AFIP or JPC between 2003 and 2017. The diagnostic terminology for surgical lung biopsies with non-neoplastic pulmonary pathology is challenging as there may be multiple patterns or diagnoses in a single specimen. We used a coding system that we have previously employed based on the text by Katzenstein, [10] which allows for up to two unique diagnostic non-neoplastic codes per surgical specimen, [11] and both codes were included in the data analysis (i.e., there are 77 coded diagnoses for the 52 surgical lung biopsy samples in this cohort).

### Statistical analysis

To address our primary objective, we qualitatively analyze retained lung particles in biopsies from DSMs and NDSMs with varied exposures and diagnoses. Additionally, we evaluated statistical associations, where sample size allowed, of service member and biopsy site characteristics with total and type-specific particle counts. These analyses included particle types that might explain differences in total particle counts in DSMs versus NDSMs. We used multivariable linear mixed effects models of each particle count outcome (log-transformed to normalize residuals) as the dependent variable; and DSM (versus NDSM), smoking status, service branch (Army versus others), and biopsy site (right versus left side, and middle or upper versus lower lobe) as independent variables, with a random intercept specified for service member (S1 Table). We hypothesized DSM, smoker status, and Army service branch as risk factors for higher particle counts, with biopsy site characteristics included as confounders. This mixed-effects model may be interpreted as an extension of the traditional linear regression model, with the random effect included to accommodate non-independence of multiple biopsy sites within individuals, and regression estimates analogous to those of the traditional regression model. Analyses were performed using the R (Vienna, Austria) lme4 package [12] with p values obtained using the lmerTest package [13]. Spearman’s correlations were additionally reported to describe the relationships of total particle counts with age at biopsy and length of deployment.

## Results

### Particle analysis

#### Chemistry

**Fig 1:**
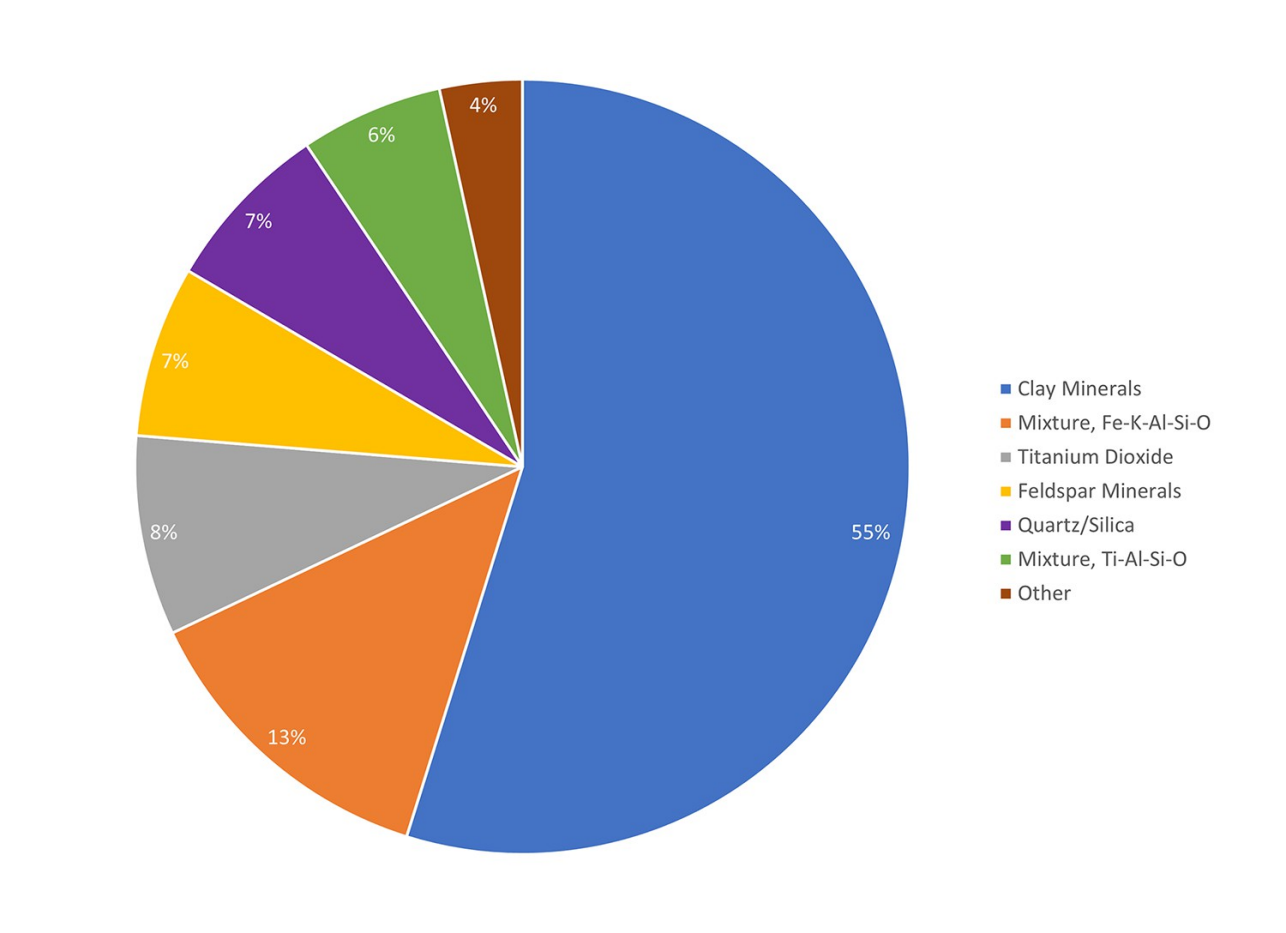
Overall particle group distribution: Particle counts. Fractions of total particles (from all samples, n = 52) for each major particle group. About 95% of all documented particles came from six main particle groups: clays, feldspars, quartz/silica, titanium dioxide, and two mixture groups (both including aluminosilicates, most likely clays).

We characterized 108,063 particles in surgical lung biopsy specimens from 52 patients: 55,737 particles from the 25 DSM biopsies and 52,326 particles from the 27 NDSM biopsies. Most of the particles (Fig 1) were clay minerals (illite, kaolinite, chlorite, and talc; K±Fe±Mg±Al-Si-O), feldspar minerals (K±Na±Ca-Al-Si-O), quartz/silica (Si-O), titanium dioxide or rutile (Ti-O), aluminum oxides (Al-O), zirconium species (Zr-O or Zr-Si-O), cerium oxide (Ce-O), iron-rich mixtures (Fe±K±Al-Si-O), and titanium-rich mixtures (Ti-Al-Si-O). The mixtures do not seem to correspond to a mineral but likely represent ultrafine or clumped particles that yield overlapping signals, most likely including aluminosilicates/clays. See the S2 File for representative examples of the various particle spectra.

The clay mineral group was composed of four components: illite, kaolinite, chlorite, and talc. Illite, kaolinite, and chlorite are all aluminosilicate (Al-Si-O) minerals that contain varying amounts of iron (Fe), magnesium (Mg), and potassium (K). Talc is a magnesium silicate (Mg-Si-O). Of these, illite, kaolinite, and chlorite are considered geological in origin, and talc is likely industrial or anthropogenic. Talc is not a known component of desert sands from the deployment region [14]. Talc is not morphologically compatible with being of the same geologic origin as illite, kaolinite, or chlorite. The overall mineral chemistry of the dataset is not compatible with being weathering products of talc-bearing rocks; we found no mafic (Mg- and/or Fe-rich) minerals that would have the same geological source as talc [15].

The feldspar mineral group consists of two main subgroups, the alkali feldspars (potassium [K]- sodium [Na]) and the plagioclase feldspars (calcium [Ca]-Na). Alkali feldspars occurred at approximately twice the frequency of plagioclase. The feldspar group had a relatively high proportion of particles that were greater than 2.5 μm in length. They also made up a large proportion of the overall particle area compared to their particle count.

Frequently occurring cerium oxide (Ce-O) particles, and to a much lesser extent, lanthanum-cerium oxide (La-Ce-O) and neodymium-cerium oxide (Nd-Ce-O and La-Ce-Nd-O) particles, are most likely of industrial origin. These are not monazite (rare earth element, [REE]-phosphate [PO_4_]) grains, as they clearly lack a phosphorus (P) peak. Monazite grain particles do occur with regularity albeit in low numbers, and these are likely geologic in origin. Zircon grains (ZrSiO_4_) occur with a similar frequency to monazite. They are distinct from zirconium oxide (Zr-O) particles and are geologic in origin. Zr-O particles occurred in many of the samples albeit in small numbers. They often show a small cerium (Ce) peak in their spectra (Zr-Ce-O particles). These are of industrial or anthropogenic origin.

Other frequently occurring metals are antimony oxides (Sb-O) and tin oxides (Sn-O), which are likely industrial or anthropogenic in origin. More uncommon metal or metal oxide particles included cadmium (Cd), lead (Pb) and lead-tin-antimony (Pb-Sn-Sb) mixtures; a single NDSM biopsy had tin-barium-lead (Sn-Ba-Pb) particles. Other uncommon particles included copper (Cu), zinc (Zn) and zinc sulfides (Zn-S-O), thorium (Th), and uranium (U). The latter occurred as single Th particles in three NDSMs and as a single U particle in one NDSM.

Particles classified as steel were found in many of the samples although never in high numbers. Steel particles have EDS spectra that generally show iron (Fe), chromium (Cr), and nickel (Ni) and may include manganese (Mn) and vanadium (V). The spectra may also show background peaks of aluminum (Al), silicon (Si), and oxygen (O) (Fe-Cr-Ni±V±Mn-Al-Si-O).

#### Morphology

Most of the particles analyzed measured less than 1 μm in greatest dimension (S2 Fig). Particles between 2.5 and 10 μm (PM_2.5–10_) contributed to most of the total particle area. Few particles were larger than 10 μm (PM_10_). Most samples had no particles of this size. Metals had the largest proportion of particles that were smaller than 0.5 μm, while feldspars and talc had the largest proportion of particles that were larger than 2.5 μm (S3 Fig).

Most particles had aspect ratios between 1:2 and 1:3 (72%). Only about 6% of all particles had an aspect ratio greater than 1:3, similar to elongate mineral particles (EMP) [16]. Most particles were roughly equant or sub-equant in aspect ratio (S4 Fig), with feldspars, talc, and the geologic clays having the highest aspect ratio (the clays may be partially explained by the clustering phenomena described below). Only seven of 52 samples (from 2 DSMs and 5 NDSMs) had a large amount (> 10%) of EMP in any of the particle groups besides talc, and all were due to feldspars. No individual sample had a large amount (> 10%) of total particles that were EMP. Aspect ratios were measured from SEM images in one plane in the tissue. Talc, for example, may be plate-like if oriented 90 degrees from the position seen in tissue. The EMPs measured in this study do not necessarily have geometric features of fibrous minerals.

**Fig 2:**
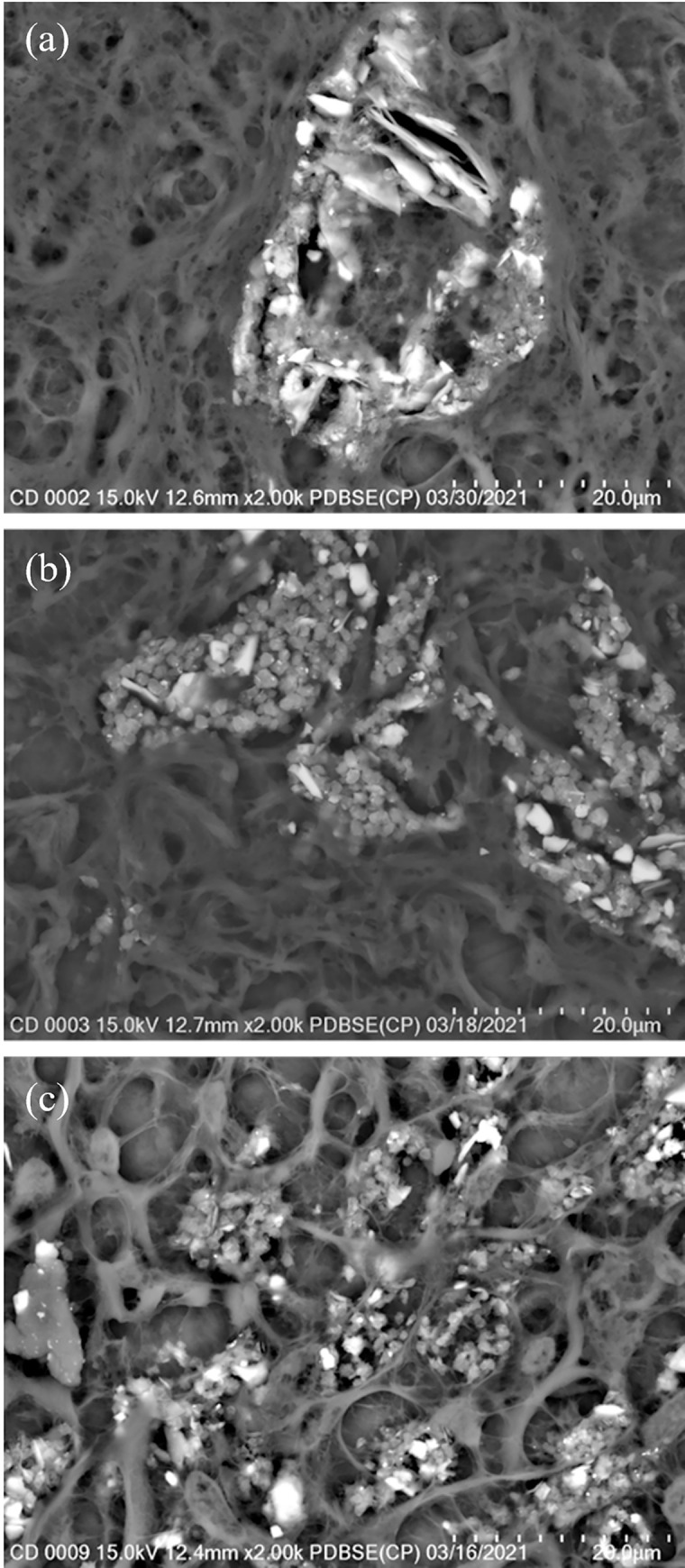
SEM images of examples of cluster types. All images are at 2000x magnification. (a) Elliptical cluster; (b) blocky clusters; (c) smaller clusters distributed throughout lung tissue.

Particulate matter in lung tissue exists mainly as clusters that are often within macrophages. Three types of clusters were observed in these samples (see Fig 2). The most common was an elliptical-shaped cluster that occurred as part of larger regions of concentrated PM. The second type was a dense, blocky cluster that also occurred as part of larger regions of high PM. The third type was a smaller cluster that appeared more evenly distributed in the tissue.

### Particle count associations

#### Military service member and biopsy characteristics

We found weak correlations between total particle counts and service member age at biopsy, length of deployment, and interval between the end of deployment and biopsy; none of these relationships are statistically significant (Table 3).

**Table 3:** Relationships between total particle count and service member age, deployment length, and deployment biopsy interval.

Relationship	Spearman’s Correlation	
	r	p
**Total particle count and age at biopsy for all service members**	0.22	0.11
**Total particle count and age at biopsy for NDSMs**	0.35	0.07
**Total particle count and age at biopsy for DSMs**	0.19	0.37
**Total particle count and length of deployment**	0.26	0.22
**Total particle count and interval between end of deployment and biopsy**	0.14	0.5

#### Smoking

NDSM smokers had the highest total metal particle counts, followed by DSM non-smokers. NDSM smokers also had the highest average metal particle counts, with the other demographic groups having similar average counts (S5 Fig).

#### Site and time of biopsy

Particle totals were influenced by the site of biopsy. For biopsies from DSMs, the right upper lobe had the highest particle loads followed by the right lower for NDSMs. The right and left lower lobes had higher particle loads for NDSMs than DSMs; for other lobes, the DSMs had higher particle loads (S6 Fig).

#### Deployed service members versus non-deployed service members

**Fig 3:**
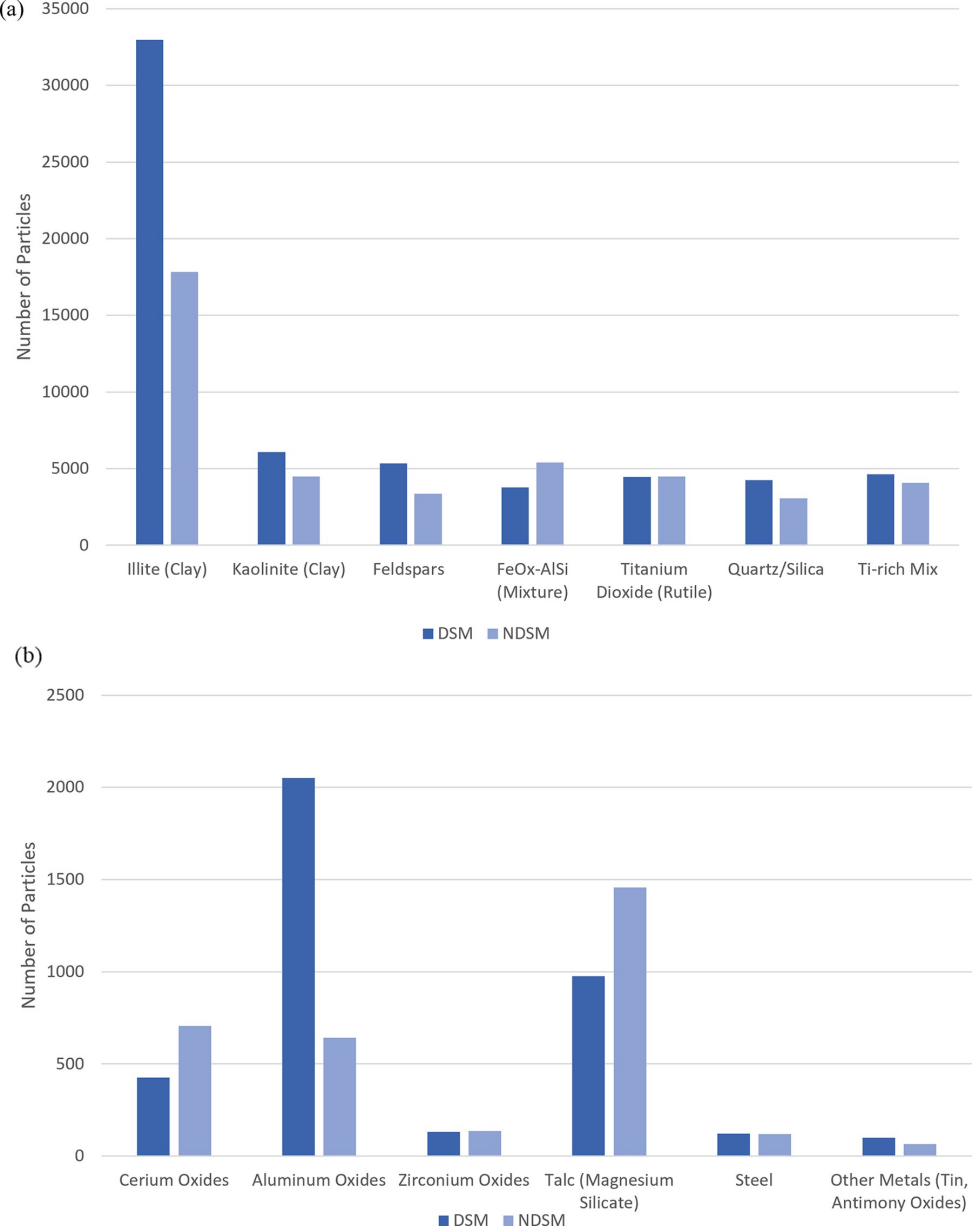
Relative abundance of particle groups in biopsies from DSMs and NDSMs. (a) Shows the most frequently occurring particle groups; (b) shows the minor particle groups.

The most frequently occurring particle groups among DSM and NDSM biopsies are shown in Fig 3. The most noticeable difference was seen for illite (clay). Among particles that are likely industrial or anthropogenic in origin, cerium oxides and talc were higher in biopsies from NDSMs. Titanium dioxide (rutile), steel, other metals, and zirconium oxides were almost equal, while aluminum oxides were more common in biopsies from DSMs, attributable to a high number of particles in a specimen from one deployed marine.

### DSM branch of service

**Fig 4:**
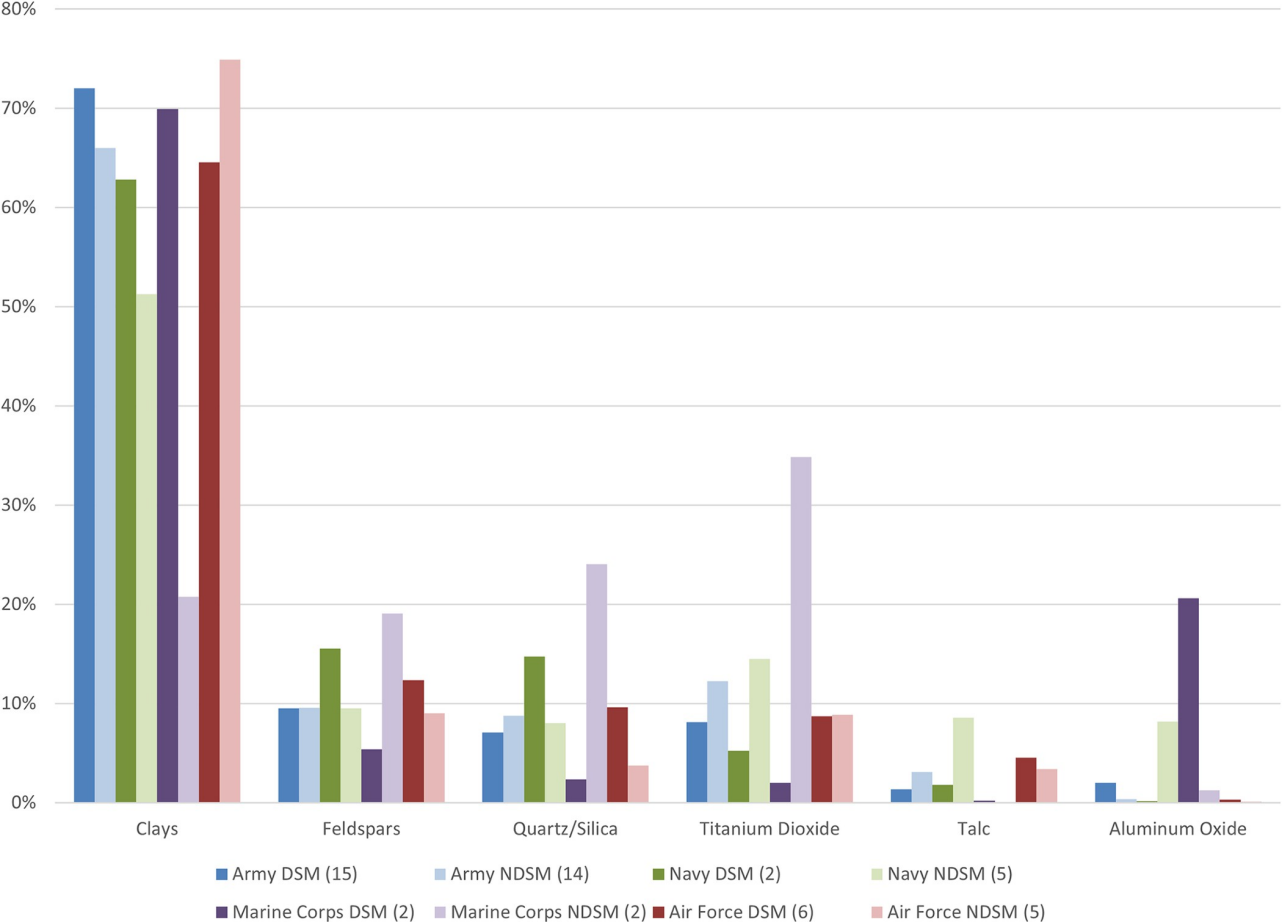
Relative frequency of particle group types across different branches of service. The number of samples for each is shown as (n). Each color represents a branch of service, and the dark/light tone represents DSM/NDSM.

The samples from Army service members (DSMs and NDSMs) had the highest particle density and comprised the largest group of samples (S7 Fig). The particle group distribution was similar for all branches of service except for NDSM Marines; their proportion of titanium dioxide and silica/quartz particles was greater than the proportion of clay particles (Fig 4). The specimens from DSM Marines had a higher proportion of aluminum oxide particles compared with those from the other services. However, there were only two service members in each Marine Corps group. The specimens from Air Force service members contained a higher proportion of clays in NDSMs compared with DSMs. Cerium oxide as a fraction of total particles was highest for Army NDSMs and DSMs (S8 Fig).

### DSM deployment region

**Fig 5:**
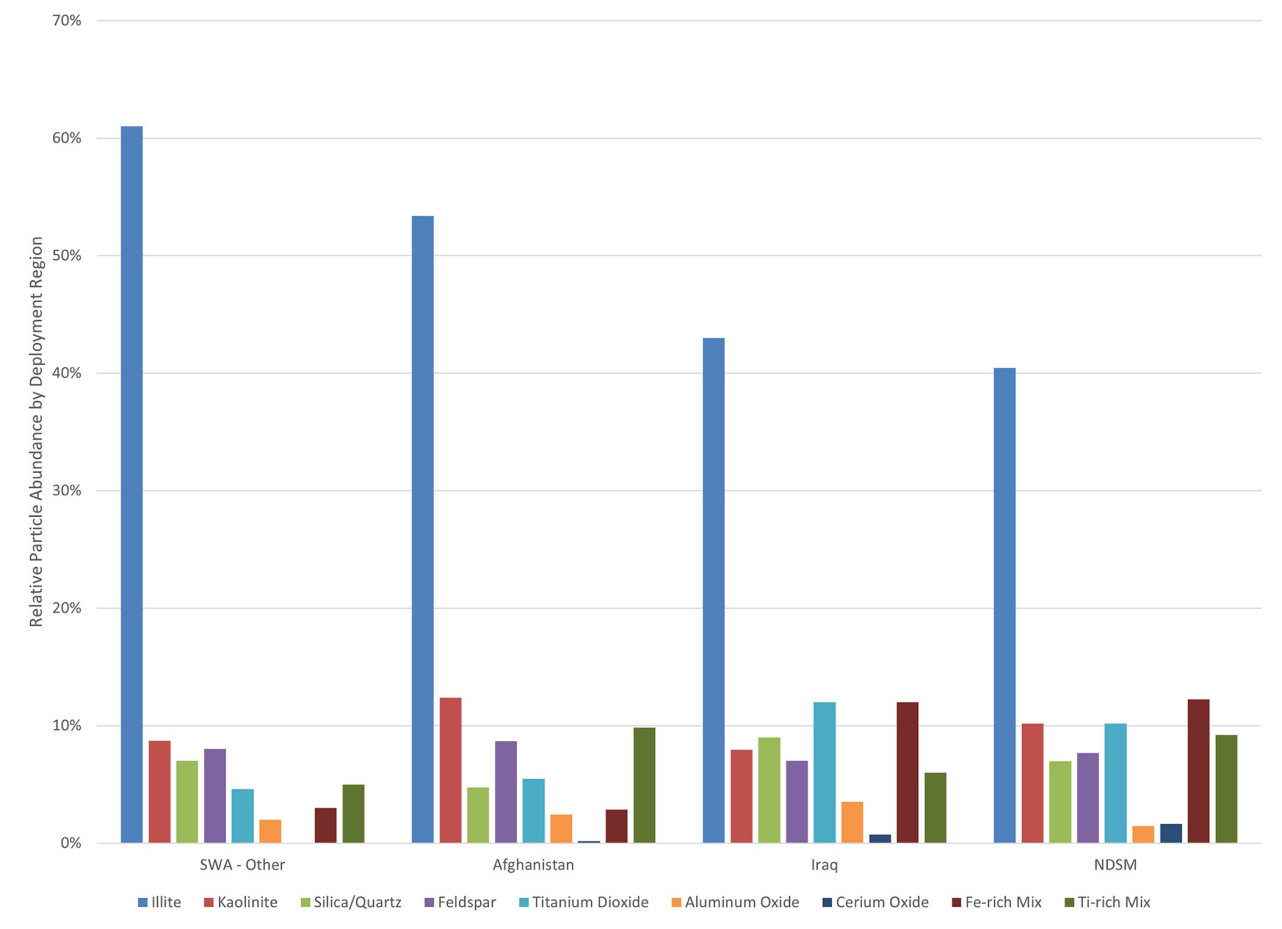
Relative particle abundance by region. Relative particle abundance across deployment regions for DSMs and overall NDSMs.

The SWA-Other group had a higher proportion of illite (clay) particles, primary components of desert sands, than the other groups (Fig 5). The SWA-Other deployment region, which was predominantly Kuwait, (n = 8) had the highest overall particle load while Afghanistan (n = 7) and Iraq (n = 10) were roughly equal. Among DSMs, the Iraq group showed the highest proportion of both cerium oxide and titanium dioxide particles. The SWA-Other group had the highest number of particles per month of deployment when factoring in the deployment length among regions. On average, the Iraq group had the finest particle size while the SWA-Other group had the coarsest particles. Iraq group DSMs were more like NDSMs with respect to titanium dioxide, cerium oxide, and iron-rich mixtures. SWA-Other and Afghanistan groups were similar with respect to higher proportions of kaolinite and feldspars with lower amounts of quartz/silica and titanium dioxide. When we analyzed particle density, taking into consideration the number of samples from each region, or normalizing the particle counts to “n,” then the Afghanistan group had a similar particle density as the Iraq group, but they were both lower than in the SWA-Other group. There was no qualitative relationship between cerium oxide and deployment length, location, or total metal particles compared with the overall particle load.

### Diagnosis

**Fig 6:**
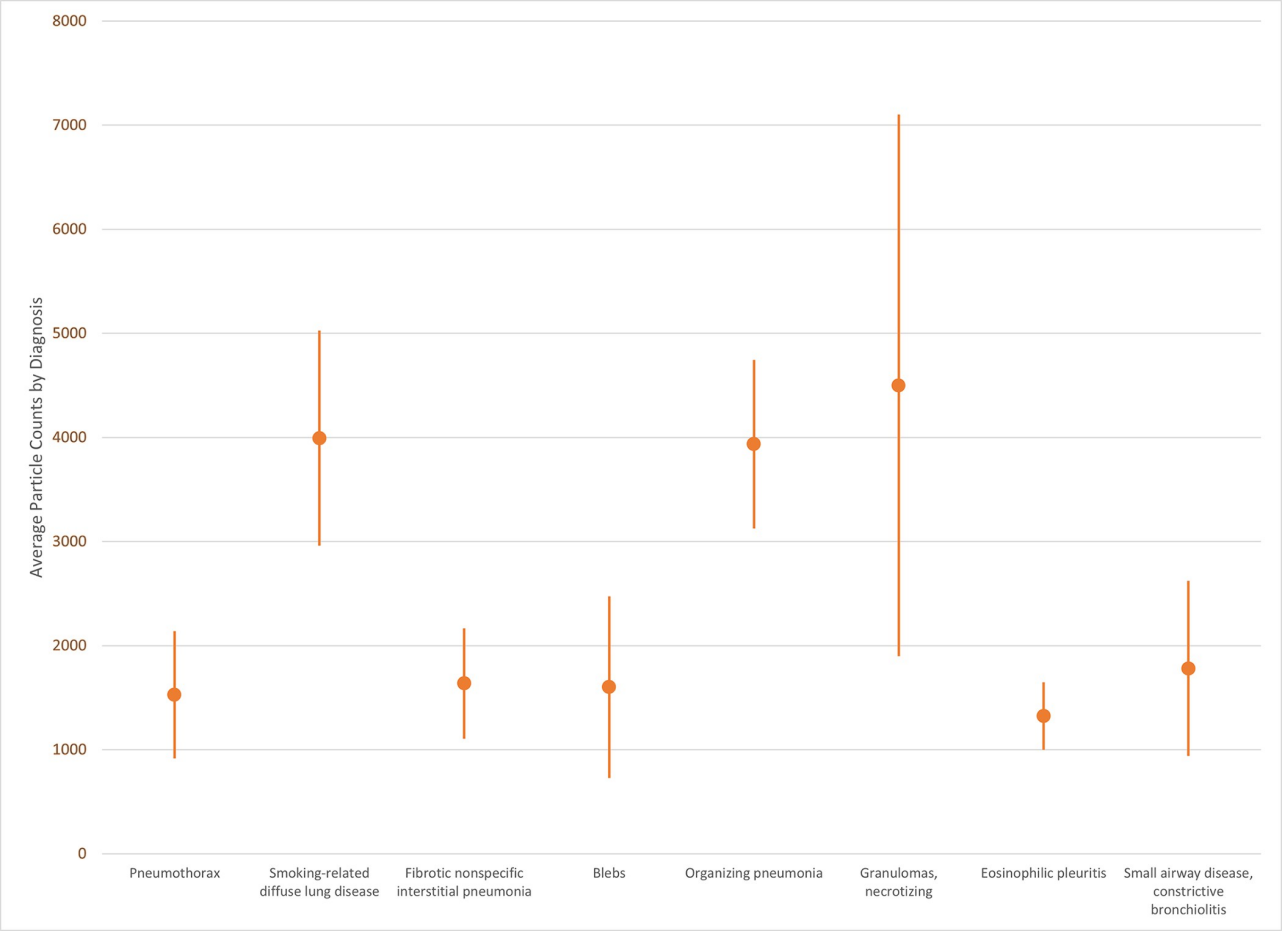
Particle distribution by diagnosis. Median particle count with median absolute deviation (MAD) error bars for the most frequently occurring diagnoses.

Smoking-related diffuse parenchymal lung disease (SRDPLD), organizing pneumonia and granulomas, necrotizing were associated with high median particle counts (Fig 6). There was a wide range in particle counts for SRDPLD and granulomas, necrotizing. The specimens from service members with pneumothorax; fibrotic nonspecific interstitial pneumonia; blebs; eosinophilic pleuritis; and small airway disease, constrictive bronchiolitis all had a similar, relatively low median particle count.

**Fig 7:**
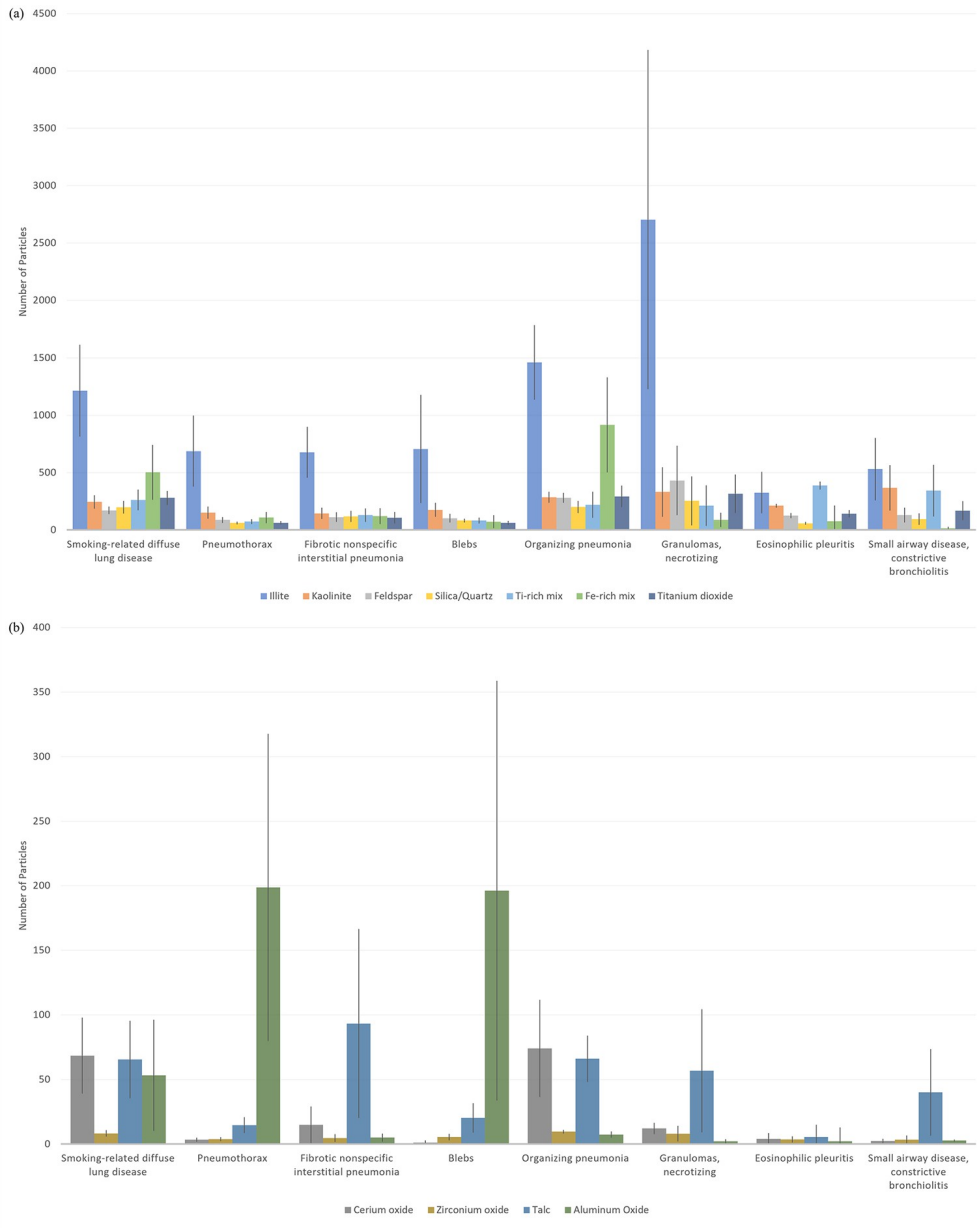
Average particle chemistry by diagnosis. Average (adjusted for “n”) (a) major and (b) minor particle group chemistry by diagnosis.

The specimens from service members with granulomas, necrotizing; SRDPLD; and organizing pneumonia had the highest number of illite particles (Fig 7); these diagnoses occurred almost equally in DSMs and NDSMs (Table 4). Service members with SRDPLD and organizing pneumonia had substantially higher numbers of iron (Fe)-rich mix particles (which may be inhaled PM with hemosiderin). The specimens from service members with eosinophilic pleuritis and small airway disease, constrictive bronchiolitis showed an unusually low amount of illite relative to the other major mineral groups when compared with the other diagnoses. Their major mineral chemical profiles were similar, with relatively high proportions of kaolinite and titanium-rich mixtures. Titanium dioxide and titanium (Ti)-rich mixtures were not elevated for any diagnosis. There was greater variation in the minor particle group chemistry among diagnoses; these particles are more likely to be of industrial origin.

**Table 4:** Diagnoses with Katzenstein-based codes for DSMs and NDSMs.

Code	Description	Total diagnosis	DSM (25 patients)	NDSM (27 patients)
0206	Smoking-related diffuse parenchymal lung disease (SRDPLD)	11	5	6
1507	Pneumothorax	10	5	5
0205	Fibrotic nonspecific interstitial pneumonia (FNSIP)	8	3	5
0211	Organizing pneumonia (OP)	6	3	3
1422	Blebs	5	4	1
0219	Granulomas, necrotizing	4	2	2
1416	Small airway disease (SAD), constrictive bronchiolitis (CB)	3	1	2
1426	Eosinophilic pleuritis	3	0	3
0220	Granulomas, non-necrotizing	2	1	1
0221	Granulomas, poorly formed airway centered (Hypersensitivity pneumonitis)	2	1	1
0902	(Infection) bacterial	2	1	1
1001	Histoplasmosis	2	1	1
1442	Small airways disease, not otherwise specified (SAD, NOS)	2	1	1
0213	Fibrosis, patchy	1	0	1
0217	Organizing pneumonia (OP) with fibrosis	1	0	1
0302	Marijuana/crack cocaine	1	0	1
0505	Eosinophilic pneumonia, chronic	1	0	1
602	Rheumatoid nodule	1	0	1
901	Infection, not otherwise specified	1	1	0
907	Human immunodeficiency virus/acquired immunodeficiency syndrome (HIV/AIDS)	1	0	1
1003	Cryptococcosis	1	1	0
1305	Pulmonary hyalinizing granuloma	1	1	0
1401	Inflammation, chronic	1	1	0
1406	Small airway injury (SAI), distortion	1	1	0
1418	Small airway disease (SAD), cellular bronchiolitis (acute, chronic inflammation)	1	1	0
1424	Acute fibrinous pleuritis	1	0	1
1425	Chronic fibrous pleuritis	1	0	1
1429	Exogenous lipoid pneumonia	1	1	0
1446	Atypical/preneoplastic/malignant not lymphoid/mass/nodule	1	1	0
1447	Empyema	1	0	1
	Totals*	**77**	**36**	**41**

The prevalence of diagnoses and associated Katzenstein-based codes [10].

*A maximum of two codes per patient were permitted.

Specimens from patients with blebs and pneumothorax showed relative abundance of aluminum oxides and similar relative abundance of other minor phases. Pneumothorax (Katzenstein-based code 1507) and blebs (1422) are related conditions and accordingly showed a similar particle profile; five patients received a 1507/1422 combination diagnosis. Fibrotic nonspecific interstitial pneumonia; granulomas, necrotizing; and small airways disease, constrictive bronchiolitis showed similar minor chemistry profiles dominated by high proportions of talc. Eosinophilic pleuritis and small airways disease, constrictive bronchiolitis varied with respect to talc despite having similar major mineral chemistry. The diagnoses SRDPLD and organizing pneumonia showed the highest number of cerium oxide particles.

### Statistical analysis

Biopsies from DSMs compared with NDSMs had statistically higher total particle counts, and higher particle counts for illite, kaolinite, feldspar, quartz/silica, and titanium (Ti)-rich mixture groups (p<0.05 for each), independent of other service member and biopsy site characteristics (S1 Table). Smoker status was not associated with total or type-specific particle counts in these multivariable models. Army service members compared with other service branches had higher quartz/silica particle counts (p = 0.02). Biopsy side (right versus left) was not associated with particle counts; however, the middle lobe (versus lower lobe) was associated with lower iron (Fe)-rich mixture group count (p = 0.03), and the upper lobe with higher titanium dioxide count (p = 0.04), with similar trends (although not statistically significant) observed for lobe site for other particle types.

## Discussion

### Particle analysis

Several methods have been applied to the analysis of PM in lung specimens. A fuller discussion of this issue is in Hayden at al. 2022 [8]. The one previous study of previously deployed patients’ lung specimens using FE-SEM/EDS-based particle analysis [17] differs from our study in several ways. We characterized over eight times the number of particles that were characterized by Lowers et al. in 2018 from 33 patients, 11 of whom were DSMs [8, 17]. We consider that our method has advantages, especially the use of routine 5-μm-thick formalin-fixed, paraffin-embedded lung surgical lung biopsy tissue sections and automation of particle detection.

#### Chemistry

The chemical compositions of particles were broadly similar between DSMs and NDSMs and were also similar to those reported by Lowers et al. [17]. Clays were the most frequent particle type, followed by feldspars, iron oxide-aluminum silicate mixtures, titanium dioxide, and quartz/silica.

The issue of clustered particles is both a feature and limitation of in situ FE-SEM/EDS analysis. While clustering within lung tissue is often due to the accumulation of particles of different chemical composition within macrophages, it may also reflect that a portion of dust particles exist as clusters prior to inhalation [18].

A case report of titanium (Ti) and iron (Fe) being present in a deployed service member’s lung biopsy was previously published [19]. However, the presence of these elements in geological and mixed particles is not unusual. It is important to distinguish between particles containing these two metallic elements and particles composed entirely of these elements. The FE-SEM/EDS technique employed in our study enables this distinction to be made quickly and reliably. Other authors have previously documented that titanium dioxide/rutile-like and Fe (rich)-containing particles are usual particulates that can be identified using SEM-based techniques in autopsy lung specimens from an adult urban US population [20]. In our study these particle types were present in lung biopsies from both DSMs and NDSMs. Neither was there a significant difference in Ti-containing particle frequency in DSMs and NDSMs, or for any individual diagnosis. The highest relative titanium dioxide particle counts were seen in Iraq DSMs and in NDSMs. There were some differences in particles that were likely of anthropogenic origin, particularly aluminum oxide, but this was largely due to the findings from one deployed Marine.

#### Morphology

One report characterized respirable particles in a dust sample from Iraq as sharp [21]. We found few particles with these characteristics in the lung biopsy tissue we analyzed. High aspect ratios were seen more often in talc particles compared to other chemical compositions (S4 Fig). Talc is not likely to have been a component of environmental dust from geological sources and may have been from manufactured or non-deployment geological origins [14, 15]. Talc particles were also present in higher numbers in biopsies from NDSMs compared with those from DSMs. Although a small proportion of particles were quartz/silica, they were predominantly of low aspect ratio and found in similar proportions in DSM and NDSM biopsies. In our institutional experience, no cases of silicosis related to deployment have been reported during the GWOT, and we are unaware of published reports to date that have supported that association. Most particles were round or sub-round across all particle types/phases. Elongate mineral particles (EMPs) were rare and were present in the biopsies from both DSMs and NDSMs. Many naturally occurring minerals have a small proportion of particles with aspect ratios higher than 3:1 [16]. We did not detect a morphological difference between the inorganic particles in DSM and NDSM biopsies. There was also no morphological difference among the particles found in biopsies across the diagnoses included in the study, but the number of available samples was small. Particle aspect ratio across deployment regions were strongly similar if not identical (std. dev. ≤1%), with no increase of high aspect ratio particles between locations.

#### Geological signature

**Fig 8:**
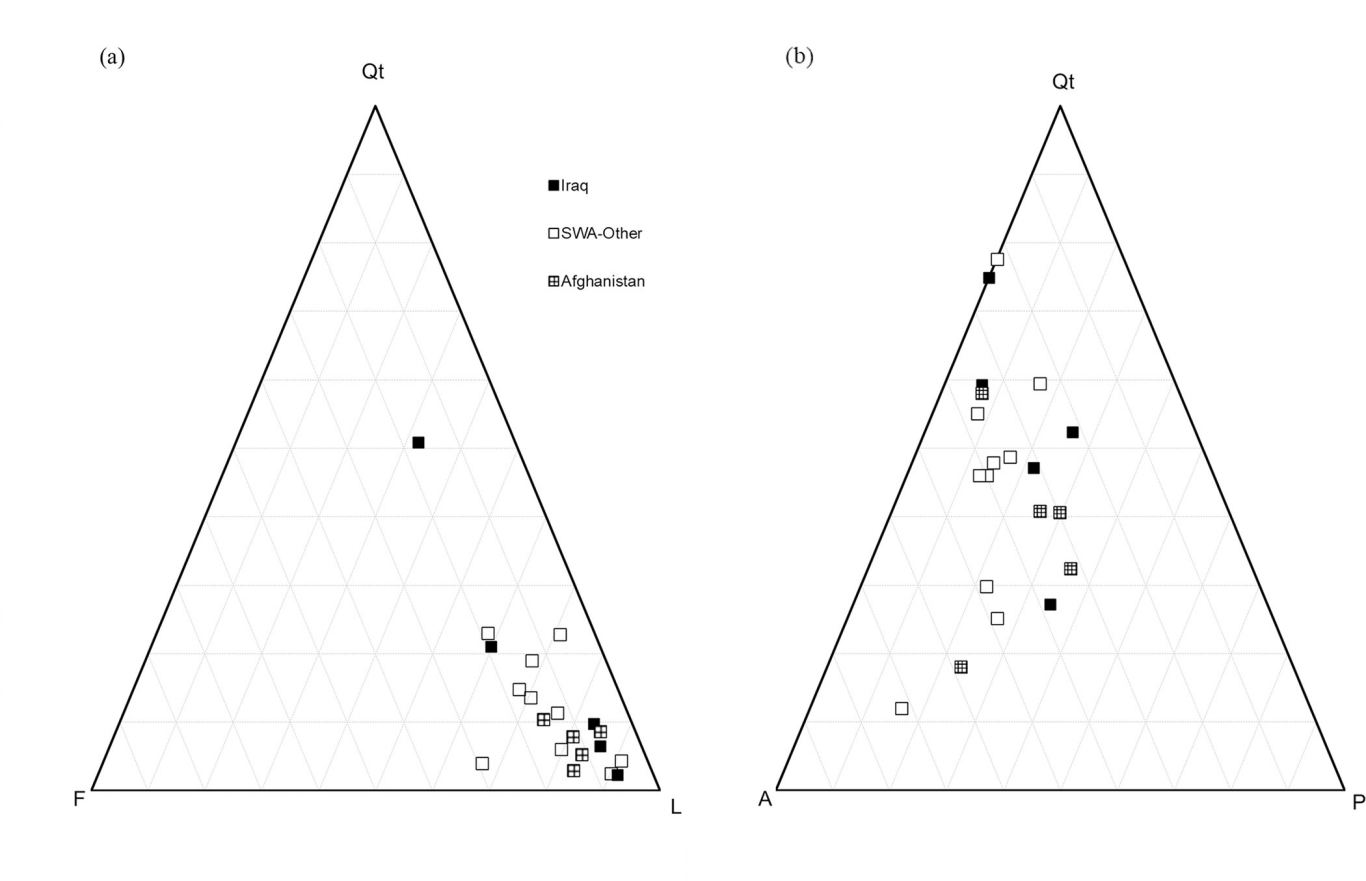
Geologic ternary plots for DSMs. (a) QtFL (quartz–feldspar–lithics) plot of DSM particles. With DSM particles dominated by clays (lithics), no distinctive grouping occurs. (b) QAP (quartz–alkali feldspar–plagioclase feldspar) plot of DSM particles. There is more variation and perhaps less inhalation or sampling bias using these particle groups.

Identifying a regional geological signature in the results proved challenging for several reasons. Many of the DSMs had a complex history of multiple deployments of various lengths to different countries. In these cases, their primary or longest deployment country was chosen for analysis. In desert regions, sandstones are often classified using a QtFL (quartz, feldspar, lithics) ternary plot [22], and this classification was applied to DSM particle chemistry. In this case, lithics are the geogenic clays chlorite, illite, and kaolinite (talc is very likely anthropogenic and thus not included). Because the particle counts of many of the samples are dominated by large numbers of clays or lithics, the data tend to cluster on the QtFL plot, and no strong trends or grouping appear (Fig 8A). The results of the QtFL plot likely show what might be described as an inhalation bias; that is, the lithics are generally finer particles than quartz/silica or feldspar and are more likely to become airborne and enter the lungs via inhalation. One would not necessarily expect that the QtFL representation of the lung tissue corresponds to a known composition of desert sands, or that a lung tissue sampling of airborne particles is fully equivalent to a hand sample of desert sands. The widest range of ternary compositions occur on a QAP plot, or quartz–alkali feldspar–plagioclase feldspar (Fig 8B). Alkali feldspars are potassium (K)- and sodium (Na)-rich end members and plagioclase feldspar is the calcium (Ca)-rich end member. These particles tend to be larger than the clays/lithics but similar in size to each other, so there may be less of a sampling bias, and this plot may better represent the environment. This type of plot may yield more well-defined mineralogical groups if more data points from biopsies from DSMs with unique regional deployment histories are obtained in future studies.

### Particle count associations with service member demographics

#### Age

We did not identify a relationship between service member age and number or type of particles. However, a decrease in particle clearance from small airways with increasing age has been reported experimentally [23], and an increase in particle accumulation with both increasing age and male sex has also been reported [20]. The DSMs and NDSMs in our study had similar mean ages (36 and 40 years, respectively), and age ranges (22–53 years and 19–54 years, respectively). These ranges were narrower than in both referenced studies [23, 20].

#### Branch of service

Within the service branches, the largest number of deployed service members were Army personnel, and they also had the longest cumulative deployment durations, reflecting the status of the entire deployed population [1, 2]. Among the biopsies from service members in all represented branches of the military, the biopsies from Army DSMs and NDSMs had the highest number of particles (S6 Fig), and the highest fraction of particles, which were cerium oxide (S7 Fig). The total number of particles may reflect both the length of deployment and Army garrison exposure, as may the cerium oxide fraction, as these findings may be related to exposure to diesel fuel, which has contained cerium oxide as an additive.

#### Deployment to biopsy interval

Although the biopsy specimens with the most particles may be expected to have been obtained from service members with the longest total duration of deployment and shortest interval between the end of last deployment and biopsy, we found weak correlations in the DSM specimens examined (Table 3). This may reflect the small number of DSMs in the study.

#### Smoking

Cigarette smoking has declined among service members over recent years, from 24% in 2011 to 13.8% in 2015 [24]. However, exposure to other potentially hazardous agents such as e-cigarettes (12.4%) and dual product use (4.7%) has increased [24]. Smokeless tobacco use by service members has also been reported to be more likely with deployment, multiple deployments, and service in a combat unit during deployment [25]. Increase in cigarette smoking initiation and recidivism has been reported to be associated with deployment, prolonged deployments, multiple deployments, and combat exposure [26].

The 32% of DSM biopsies with histological features related to smoking in our cohort is higher than the prevalence of cigarette smoking reported for the total US military population. Smoking is likely to be overrepresented in those undergoing surgical lung biopsy due to its adverse effects on pulmonary health among both DSMs and NDSMs.

#### Diagnoses

This study reports the particle analysis results from 25 biopsies from DSMs. Among these service members there were 36 unique diagnoses when a maximum of two diagnoses per biopsy were allowed. The most frequent DSM diagnoses were smoking-related diffuse parenchymal lung disease (5), spontaneous pneumothorax (5), and blebs (4). It is difficult to ascertain with such low numbers of individual diagnoses whether retained inorganic particulates or subsets of particles are associated with specific diagnoses. However, some trends appeared. For example, the “iron-rich” particle category was most prominent in patients with SRDPLD and OP. The former is smoking-related and histologically features hemosiderin-laden macrophages; the latter probably represents the sequelae of focal hemorrhage.

Illite was the most abundant particle type in both DSMs and NDSMs (Fig 3). The diagnoses with the largest number of illite particles were SRDPLD, in 11 service members (5 DSMs and 6 NDSMs); organizing pneumonia in six (3 DSMS and 3 NDSMs); and granulomas, necrotizing in four (2 DSMs and 2 NDSMs) (Table 4, Fig 7). These three diagnoses are commonly associated with smoking, prior infection, and infection, respectively. The relationship between agricultural dust exposure (including aluminum silicates) and veterinary pathology with (silicate) pneumoconiosis has been examined in publications based on asymptomatic autopsy and necropsy studies, [9, 27]. While inhaled illite may have a role in lung (small airway) inflammation and eventual fibrosis, it is often present mixed with a smaller proportion of quartz/silica, and in the case of agricultural work may also be associated with inhaled organic material and smoke inhalation (tobacco or burning biomass). The service members in this study underwent surgical biopsy for work up of symptomatic and radiologic clinical presentations that resulted in the diagnoses made. While these data do not exclude a contribution of illite to the pulmonary pathology reported, ascribing the pulmonary diagnoses in the current study to the presence of illite would be speculative.

One DSM and two NDSMs had a diagnosis of small airways disease, constrictive bronchiolitis. One DSM and one NDSM had a diagnosis of small airways disease, not otherwise specified. Two DSMs had other small airways diagnoses. The case series published by King et al. [28] focused attention on the development of non-neoplastic lung diseases, particularly constrictive bronchiolitis (CB), in formerly deployed soldiers. Those diagnosed with CB using video-assisted thoracoscopic (VATS) lung biopsies included a high proportion (28 of 38; 74%) who reported exposure to a sulfur mine fire, which is a potential source for the development of CB. An earlier publication addressing pulmonary pathology diagnoses made between 2002 and 2015 at the former AFIP and JPC recorded a far smaller proportion of CB diagnoses in a larger review of pulmonary pathology from DSMs [11]. The former AFIP/JPC observations have found some support from pulmonary pathology publications (mainly case reports and case series) that have broadened the pathological diagnoses and findings associated with deployment since 2011 [19, 29–31].

Chronic exposure to high levels of PM has been reported to be associated with small airways remodeling in an autopsy study of never-smoking, urban-dwelling women with no occupational exposure history [32]. It has recently been estimated that between 2000 and 2019, almost the entire global population was exposed to PM_2.5_ levels that exceeded the 2021 WHO annual limit [33]. The human health consequences of this reported environmental exposure may not be evident for many years. Developing associations between specific non-neoplastic pulmonary diseases and high PM exposure should involve both prolonged and detailed environmental monitoring and rigorous medical documentation of diagnoses.

The absence of reports of deployment-related silicosis to date indicates that this occupational lung disease does not appear to be related to GWOT deployment.

### Limitations of the study

The interpretation of these study results is limited by several statistical and other methodologic factors. We sought to characterize qualitative relationships of DSM and NDSM patients and service characteristics with detailed particle analysis in lung biopsies from this unique cohort. However, inferences from these descriptive analyses are limited by the small numbers of samples available for several strata of interest, including region of deployment, diagnosis, and smoking status. Where sample size allowed, we used a statistical approach to estimate associations of deployment status and patient characteristics with total and type-specific particle counts. However, selected confounders included in these models were limited to several key available measures to avoid model over-fitting.

Availability of surgical lung biopsy specimens for analysis is dependent on paraffin blocks being available in the JPC tissue repository. The biopsies were not pre-selected based on patients’ diagnoses. Most diagnoses were present in both the DSMs and NDSMs. In future studies it may be of particular interest to try to enlarge this cohort and enrich for diagnoses such as small airways disease, constrictive bronchiolitis.

Inter-observer variation in non-neoplastic pulmonary diagnoses from surgical lung biopsies has been reported [34]. Diagnostic misclassification has the potential to influence conclusions about the specificity of associations between diagnoses and exposures experienced during deployment. The quality of diagnoses reported in this study is high, as they were made at the former AFIP and the JPC by subspecialized expert pulmonary pathologists performing second opinion consultations.

Smoking histories were incomplete, and smoking does contribute to both neoplastic and non-neoplastic pulmonary pathology. The introduction of newer inhaled products such as e-cigarettes may also complicate how accurately smoking histories are documented. Using reported histologic features to determine smoking category may introduce errors but was considered preferable to reliance on incomplete historical records.

The particle types characterized in this study were inorganic and had a lower size limit of 170 nm. FE-SEM/EDS can detect smaller particles (nanoparticles), which may have a role in the pathogenesis of non-neoplastic lung disease via oxidative stress and inflammation [35]. This may be an interesting area for additional research. Carbonaceous particles (including combustion products) were not examined, as they have similar composition to the background tissue and carbon substrate.

Finally, the FE-SEM/EDS technique employed is a qualitative measurement of the inorganic retained particle burden [8]. It accurately records the morphology and chemical composition of retained inorganic particles, over a constant total area of lung (1 x 1 mm) and at a constant magnification (2,000 X). However, the results are not quantitative, as has been reported in earlier SEM-based studies [20, 36].

## Conclusions

This study is the first to report FE-SEM/EDS findings with both pulmonary diagnostic and military deployment data and includes a comparison group. PM was present in surgical lung biopsy specimens from DSMs and NDSMs. The major and minor particle types were present in both groups. DSMs had statistically higher total particle counts than NDSMs and higher particle counts for illite, kaolinite, feldspars, quartz/silica, and titanium (Ti)-rich silicate mixtures. We did not identify deployment-specific or diagnosis-specific PM in this study. The non-neoplastic pulmonary diagnoses discussed in this study may not be associated with inorganic PM directly; other possible etiologies such as exposure to volatile chemicals and the sequelae of infection are not evaluated by our FE-SEM/EDS technique. Smoking-related pulmonary diseases were present in both DSMs and NDSMs, and they are likely over-represented in those undergoing surgical lung biopsy. FE-SEM/EDS is a powerful technique for in situ inorganic particle analysis in lung tissue and could be applied in the future to analysis of nanoparticles. These results support the examination of particle-related lung disease in DSMs in the context of comparison groups, such as NDSMs, to assist in determining the strength of associations between specific pulmonary pathology diagnoses and deployment-specific exposure to inhaled inorganic particles.

## Supporting information

S1 Fig The distribution of the biopsies from the DSMs and NDSMs relative to the deployment end dates of the DSMs. (TIF)

S2 Fig Particle size distribution total. (TIF)

S3 Fig Particle size distribution by mineral groups. (TIF)

S4 Fig Aspect ratio distribution by mineral groups. (TIF)

S5 Fig Total and average (normalized to n) metal particles as they relate to deployment and smoking status. (TIF)

S6 Fig Total particles by lobe (a) and average total particles by lobe per patient (b).
(TIF)

S7 Fig Particle density by branch of service. Each bar represents a patient.
(TIF)

S8 Fig Cerium Oxide as a fraction of total particles. Each bar represents a patient.
(TIF)

S1 Table Estimated associations of service member and biopsy site characteristics with total and type-specific particle counts. Estimates are from separate multivariable linear mixed effects models of the log-transformed outcome as the dependent variable and all characteristics as independent variables with a random intercept for service member. SE = standard error. Other service branches = Navy, Marine Corps, or Air Force.
(DOCX)

S1 File Dataset to support analysis. (XLSX)

S2 File Representative EDS spectra. (PDF)

## References

[1] Defense Manpower Data Center, Monterey CA. Lewin-Smith M. Personal communication. December 2022.

[2] Wenger JW O’Connell C, Cottrell L. Examination of recent deployment experience across the services and components. Santa Monica, CA: Rand Corporation; 2018.

[3] Institute of Medicine. Long-term health consequences of exposure to burn pits in Iraq and Afghanistan. Washington, DC: National Academies Press; 2011.

[4] Rose CR. Military service and lung disease. Clin Chest Med. 2012;33(4):705–714. doi: 10.1016/j.ccm.2012.09.001 23153610

[5] Falvo MJ, Bradley M, Brooks SM. Is deployment an “exposure” in military personnel? Occup Med Forum, JOEM. 2014. doi: 10.1097/JOM.0b013e3182942a43 24423703

[6] Garshick E, Abraham JH, Baird CP, et al. Respiratory health after military service in Southwest Asia and Afghanistan. An official American Thoracic Society workshop report: Executive summary. Ann Am Thorac Soc. 2019;16(8):937–946. 10.1513/AnnalsATS.201904-344WS PMC6774741 31368802

[7] . Strausborger SL, Jenkins HM, Franks TJ, Lewin-Smith MR. Particle analysis by scanning electron microscopy with energy dispersive X-ray analysis in pulmonary pathology specimens from US military service members deployed during the Global War on Terror (GWOT) 2002–2015. Mil Med. 2021Jan 25;186(Suppl 1):784–788. doi: 10.1093/milmed/usaa318 33499496

[8] Hayden LA, Lewin-Smith M, Strausborger S. Automated particle analysis using field-emission scanning electron microscopy (FE-SEM) and energy dispersive X-ray spectroscopy (EDS) to characterize inhaled particulate matter (PM) in biopsied lung tissue. Microsc Microanal. 2022 Dec 20:1–9. doi: 10.1093/mic/mic/ozac015/6927143

[9] Schenker MB, Pinkerton KE, Mitchell D et al. Pneumoconiosis from agricultural dust exposure among young California farmworkers. Environ Health Perspect. 2009;117(6):988–994. doi: 10.1289/ehp.0800144 19590695 PMC2702418

[10] Katzenstein A-LA, editor. Katzenstein and Askin’s Surgical Pathology of Non-Neoplastic Lung Disease: Volume 13 in the Major Problems in Pathology Series. 4th ed. Philadelphia, PA: Elsevier Saunders; 2006. 7087547

[11] Lewin-Smith MR, Martinez A, Brooks DI, Franks TJ. Pulmonary pathology diagnoses in the US military during the Global War on Terrorism. Lung. 2021;199(4):345–355. doi: 10.1007/s00408-021-00446-6 34363506

[12] Bates D, Mächler M, Bolke B, Walker S. Fitting linear mixed-effects models using lme4. J Stat Softw. 2015;67(1).

[13] Kuznetsova A, Brockhoff PB, Christensen RHB. lmerTest package: Tests in linear mixed effects models. J Stat Softw. 2017;82(13):1–26.

[14] Benaafi M, Abdullatif OM. Sedimentological, mineralogical, and geochemical characterization of sand dunes in Saudi Arabia. Arabian J Geosci. 2015;8:11073–11092. doi: 10.1007/s12517-015-1970-9

[15] Wilson MJ. Weathering of the primary rock-forming minerals: processes, products and rates. Clay Minerals. 2004;39:233–266.

[16] Gunter ME. Elongate mineral particles in the natural environment. Toxicol Appl Pharmacol. 2018;361:157–164. doi: 10.1016/j.taap.2018.09.024 30291935

[17] Lowers HA, Breit GN, Strand M, Pillers RM, Meeker GP, Todorov TI, et al. Method to characterize inorganic particulates in lung tissue biopsies using field emission scanning electron microscopy. Toxicol Mech Methods. 2018;28:475–487. doi: 10.1080/15376516.2018.1449042 29685079

[18] Sokolik IN, Toon OB. Incorporation of mineralogical composition into models of the radiative properties of mineral aerosol from UV to IR wavelength. J Geophys Res. 1999;104(D8):9423–9444.

[19] Szema AM, Schmidt MP, Lanzirotti A, et al. Titanium and iron in lung of a soldier with nonspecific interstitial pneumonitis and bronchiolitis after returning from Iraq. J Occup Environ Med. 2012;54(1):1–2. doi: 10.1097/JOM.0b013e31824327ca 22227868

[20] Stettler LE, Platek SF, Riley RD, Mastin JP, Simon SD. Lung particulate burdens of subjects from the Cincinnati, Ohio urban area. Scanning Microsc. 1991;5:85–94. 1647057

[21] Szema AM, Reeder RJ, Harrington AD, Schmidt M, Liu J, Golightly M, et al. Iraq dust is respirable, sharp, and metal-laden and induces lung inflammation with fibrosis in mice via IL-2 upregulation and depletion of regulatory T cells. J Occup Environ Med. 2014;56:243–251. doi: 10.1097/JOM.0000000000000119 24603199 PMC4037815

[22] Zahid K, Barbeau D. Constructing sandstone provenance and classification ternary diagrams using an electronic spreadsheet. J Sed Res. 2011;81:702–707.

[23] Svartengren M, Falk R, Philipson K. Long-term clearance from small airways decreases with age. Eur Respir J. 2005;26:609–615. doi: 10.1183/09031936.05.00002105 16204590

[24] Keltner CH, Kao T-C, Ahmed A, Mancuso JD. E-cigarette and dual product use as an emerging health threat to the US military. Tob Prev Cessat. 2021 Jun;7:43. doi: 10.18332/tpc/135516 34141958 PMC8176862

[25] Lin J, Zhu K, Solivan-Ortiz AM, et al. Deployment and smokeless tobacco use among active-duty service members in the U.S. military. Mil Med. 2019;184(3/4):e183. doi: 10.1093/milmed/usy186 30085231

[26] Smith B, Ryan MAK, Wingard DL, et al. Cigarette smoking and military deployment: A prospective evaluation. Am J Prev Med. 2008;35(6):539–546. doi: 10.1016/j.amepre.2008.07.009 18842388

[27] Brambilla C, Abraham J, Brambilla E, et al. Comparative pathology of silicate pneumoconiosis. Am J Pathol. 1979;96:149–170. 223447 PMC2042347

[28] . King MS, Eisenberg R, Newman JH, et al. Constrictive bronchiolitis in soldiers returning from Iraq and Afghanistan. N Engl J Med. 2011;365:222–230. doi: 10.1056/NEJMoa1101388 21774710 PMC3296566

[29] Welsh CH, Miller YE. Denver Veterans Affairs Medical Center experience with postdeployment dyspnea case reports. In: Baird CP, Harkins DK, editors. Airborne Hazards Related to Deployment. Fort Sam Houston, TX: Borden Institute;2015. p. 213–217.

[30] Gordetsky J, Kim C, Miller RF, Mehrad M. Non-necrotizing granulomatous pneumonitis and chronic pleuritis in soldiers deployed to Southwest Asia. Histopathology. 2020;77:453–459. Available from: doi: 10.1111/his.14135 32379353

[31] Rose CR, Moore CM, Zell-Baran LM et al. Small airways and airspace inflammation and injury distinguish lung histopathology in deployed military personnel from healthy and diseased lungs. Human Pathology. 2022;124:56–66. doi: 10.1016/j.humpath.2022.02.014 35240130

[32] Churg A, Bauer M, Avila-Casado MdC, et al. Chronic exposure to high levels of particulate air pollution and small airway remodeling. Environ Health Perspect. 2003;111:714–718. doi: 10.1289/ehp.6042 12727599 PMC1241480

[33] Yu W, Ye T, Zhang Y, et al. Global estimates of daily ambient fine particulate matter concentrations and unequal spatiotemporal distribution of population exposure: a machine learning modelling study. Lancet Planet Health. 2023;7:e209–218. doi: 10.1016/S2542-5196(23)00008-6 36889862

[34] Nicholson AG, Addis BJ, Bhatucha H, et al. Inter-observer variation between pathologists in diffuse parenchymal lung disease. Thorax. 2004;59(6):500–505. doi: 10.1136/thx.2003.011734 15170033 PMC1747021

[35] Donaldson K, Stone V. Current hypotheses on the mechanisms of toxicity of ultrafine particles. Ann Ist Super Sanità. 2003;39(3):405–410. 15098562

[36] Abraham JL, Burnett BR. Quantitative analysis of inorganic particulate burden in situ in tissue sections. Scan Electron Microsc. 1983;(Pt 2):681–696. 6356333

