# Supplementary figures and images for: Particle analysis of surgical lung biopsies from deployed and non-deployed US service members during the Global War on Terrorism
Source: PLoS One. 2024 Apr 11;19(4):e0301868. doi: 10.1371/journal.pone.0301868 (PMC11008878; doi:10.1371/journal.pone.0301868)

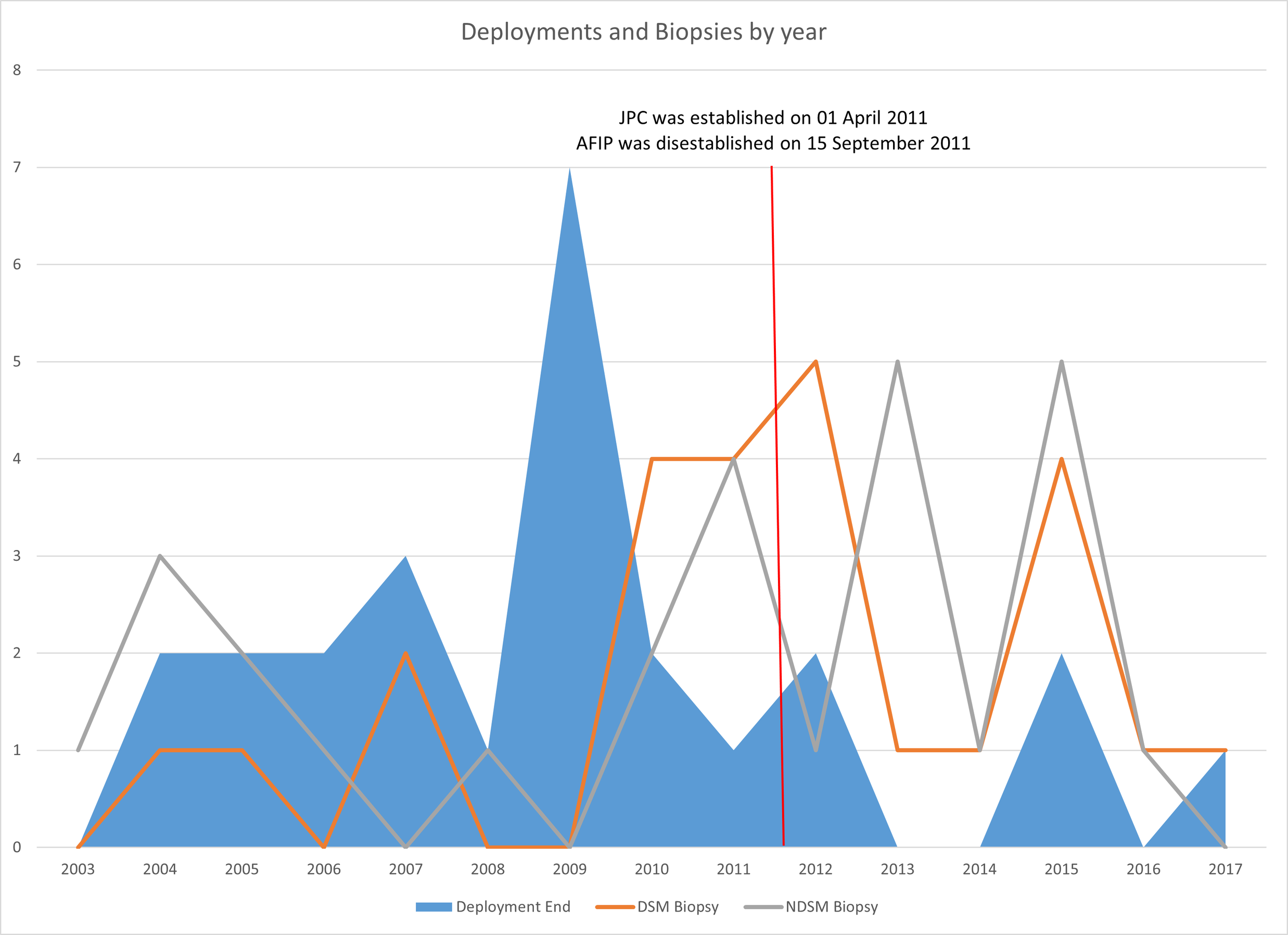

Supplement: S1 Fig — (TIF) [file pone.0301868.s001.tif]

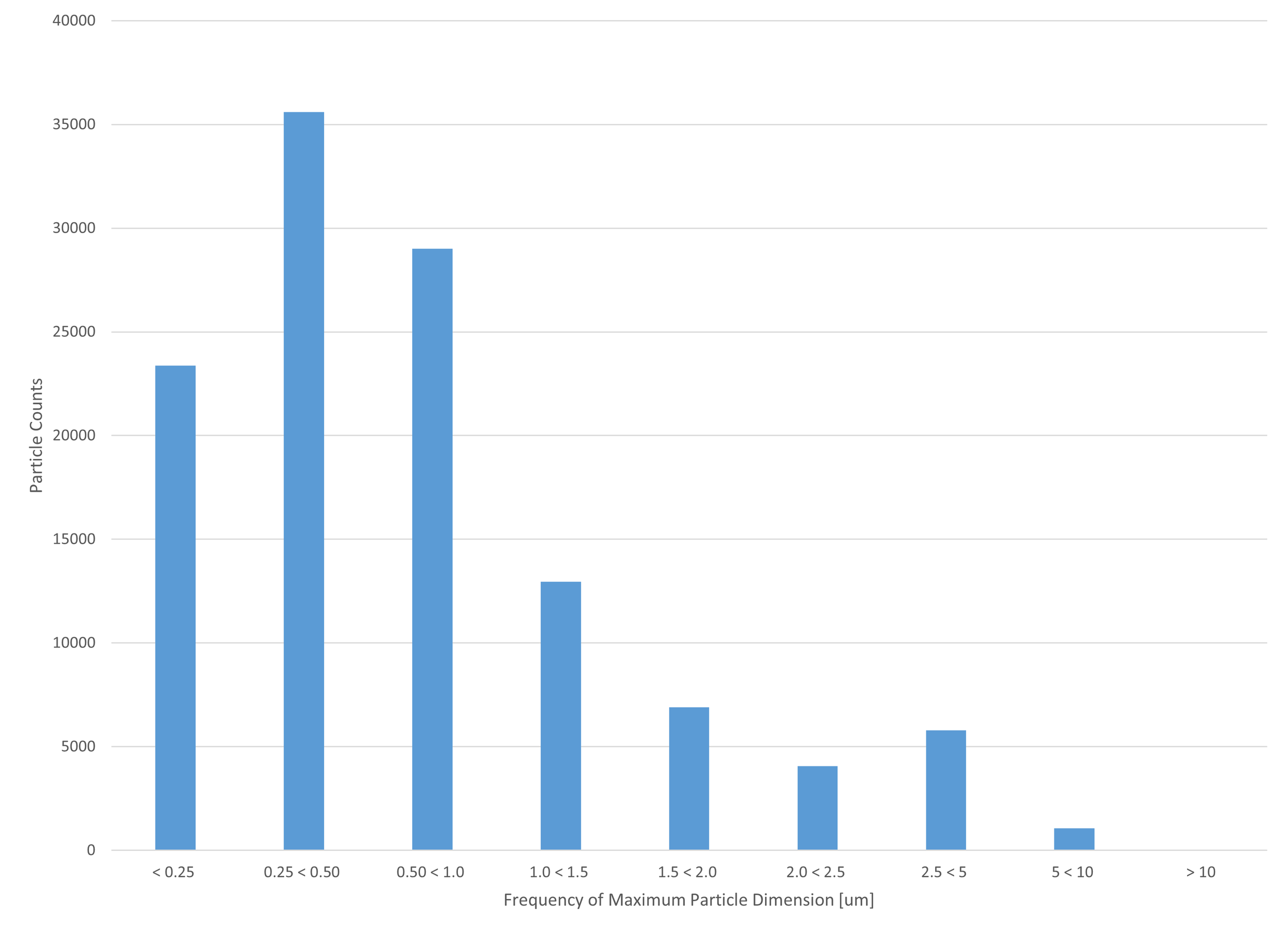

Supplement: S2 Fig — (TIF) [file pone.0301868.s002.tif]

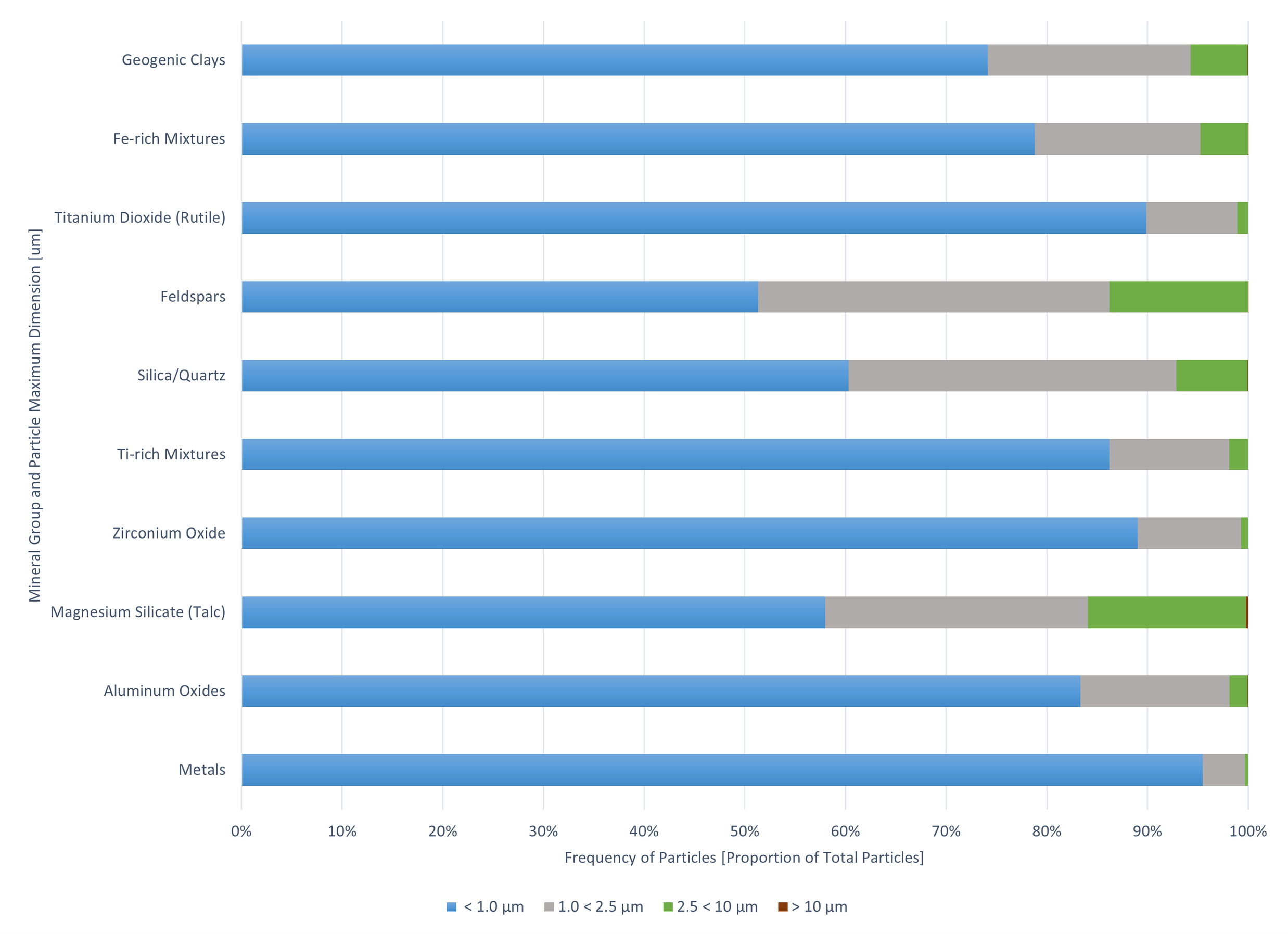

Supplement: S3 Fig — (TIF) [file pone.0301868.s003.tif]

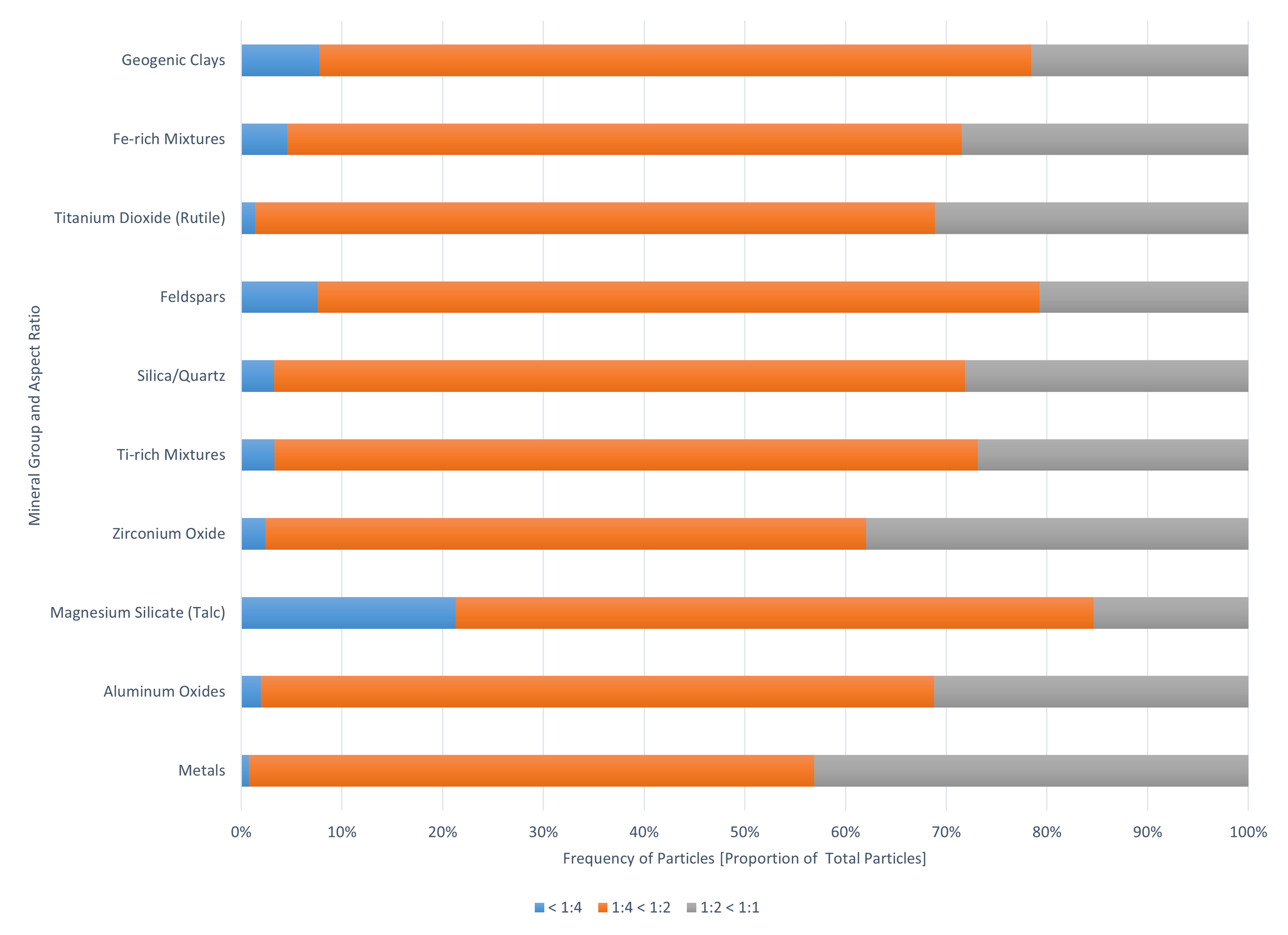

Supplement: S4 Fig — (TIF) [file pone.0301868.s004.tif]

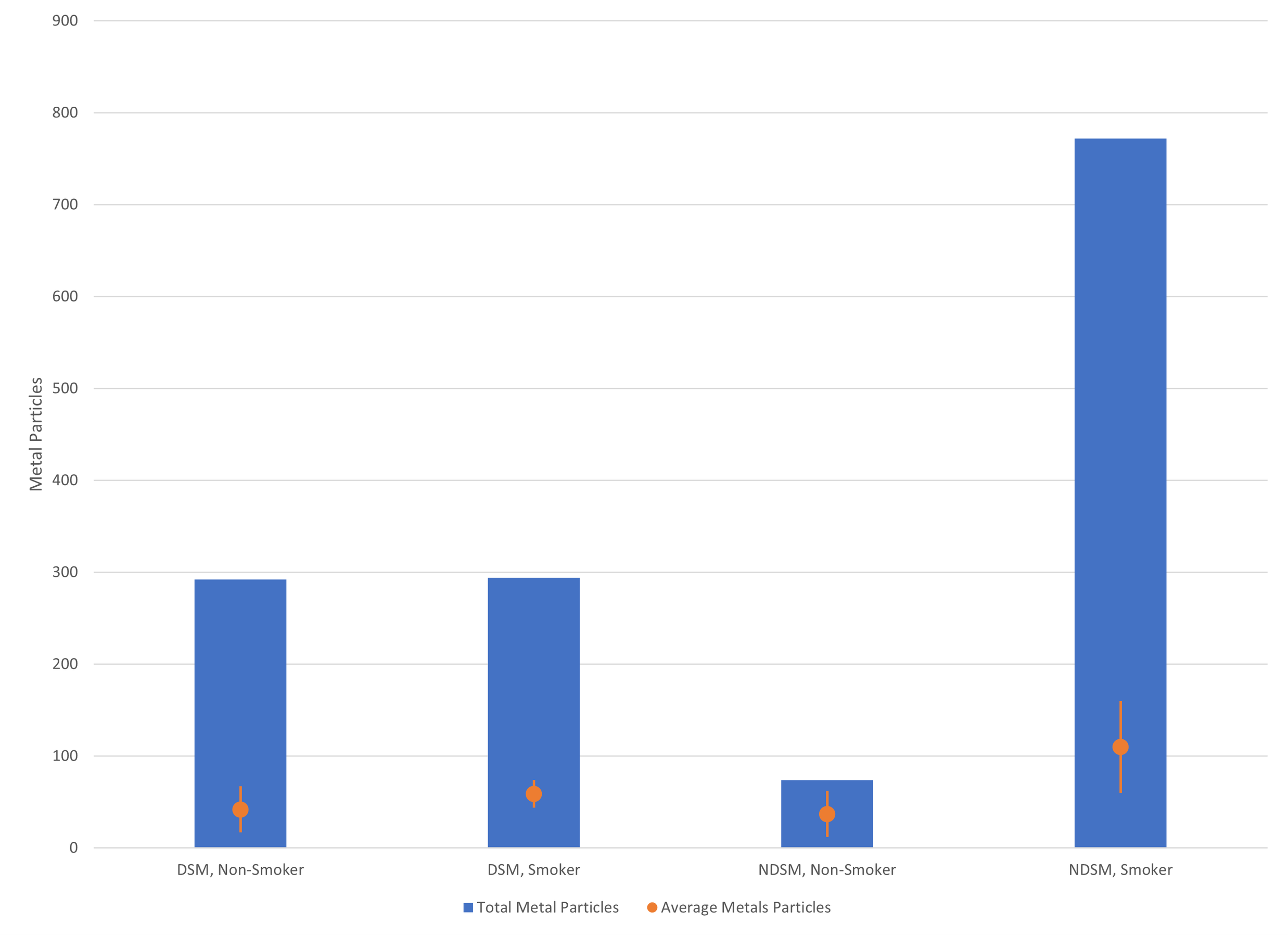

Supplement: S5 Fig — (TIF) [file pone.0301868.s005.tif]

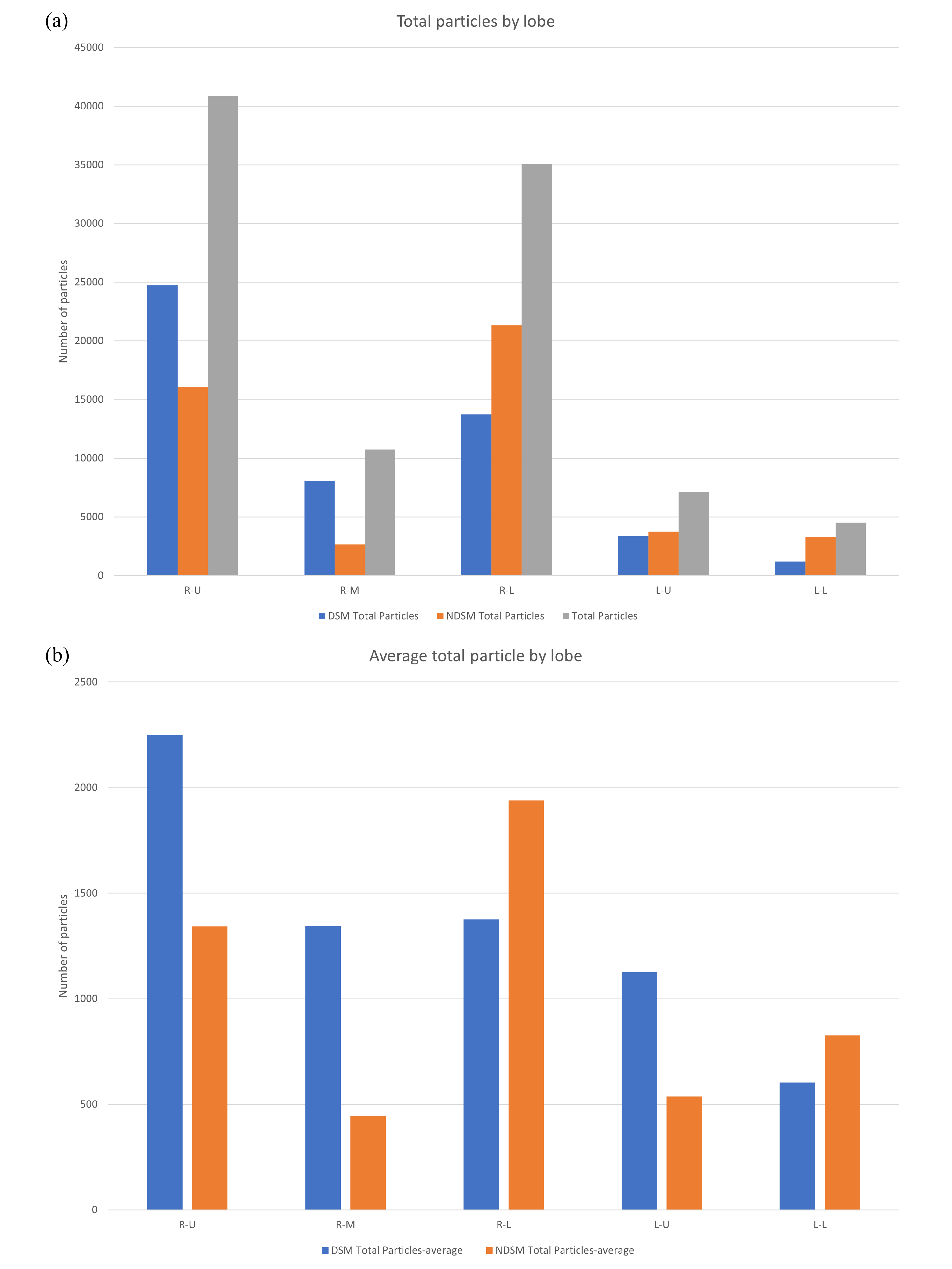

Supplement: S6 Fig — Total particles by lobe (a) and average total particles by lobe per patient (b). (TIF) [file pone.0301868.s006.TIF]

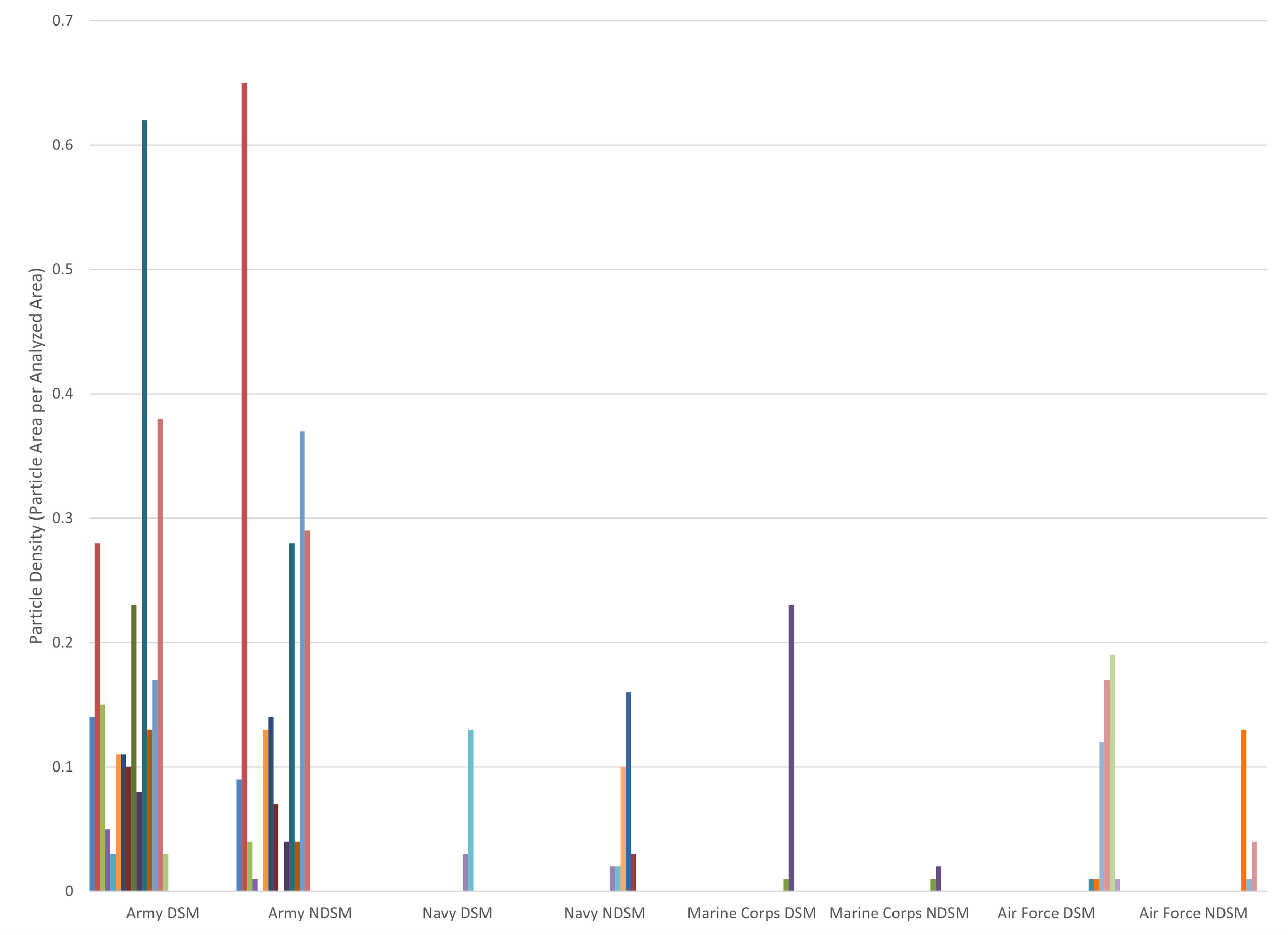

Supplement: S7 Fig — Each bar represents a patient. (TIF) [file pone.0301868.s007.tif]

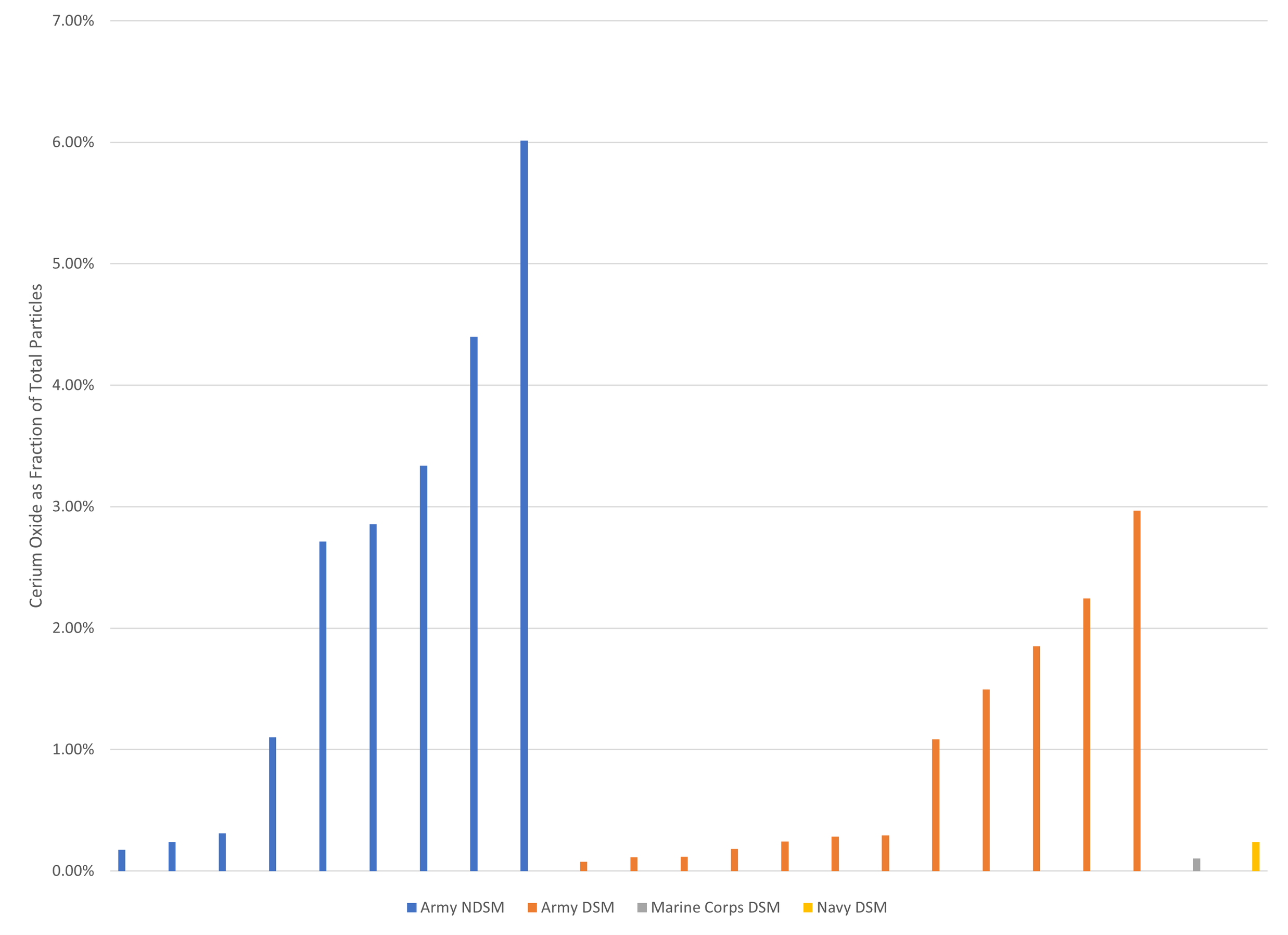

Supplement: S8 Fig — Each bar represents a patient. (TIF) [file pone.0301868.s008.tif]

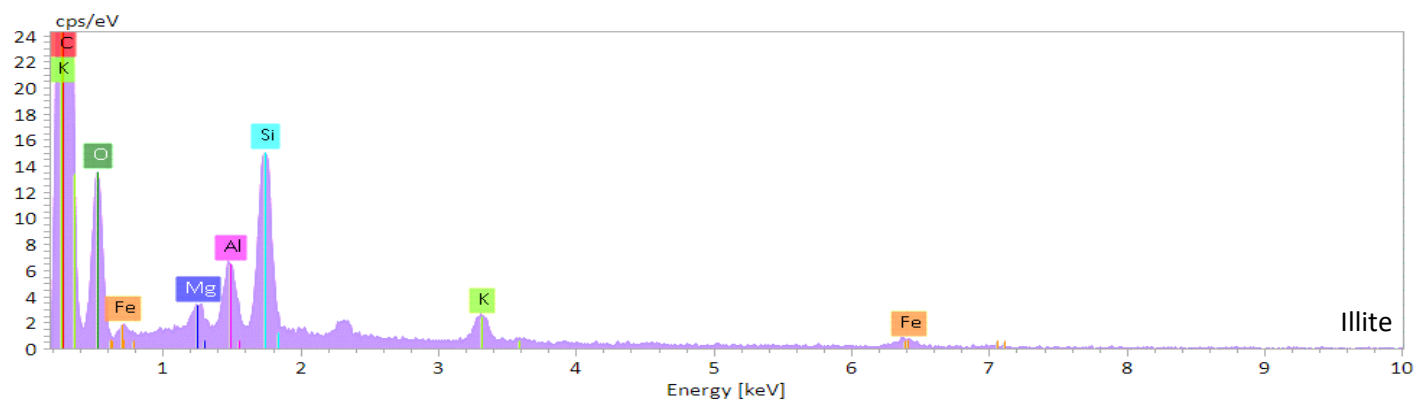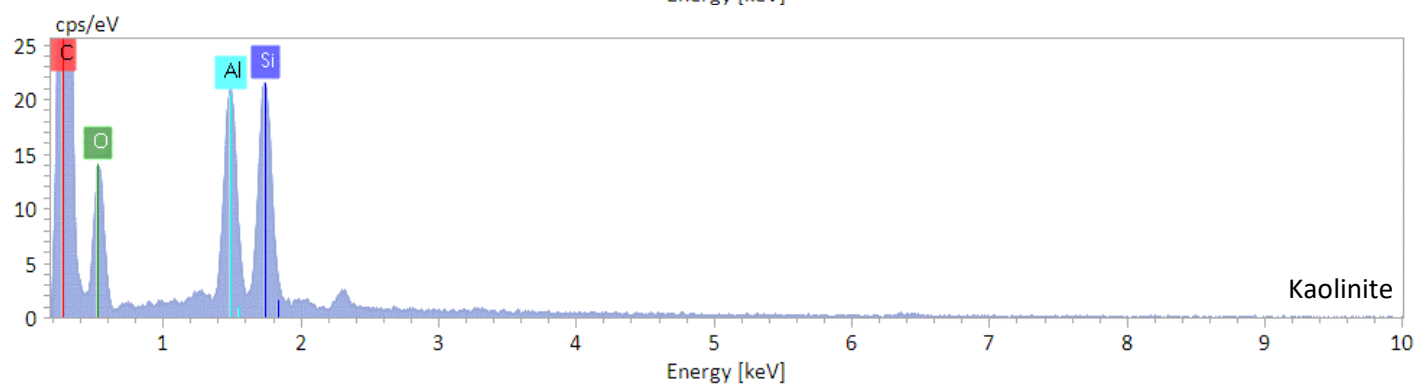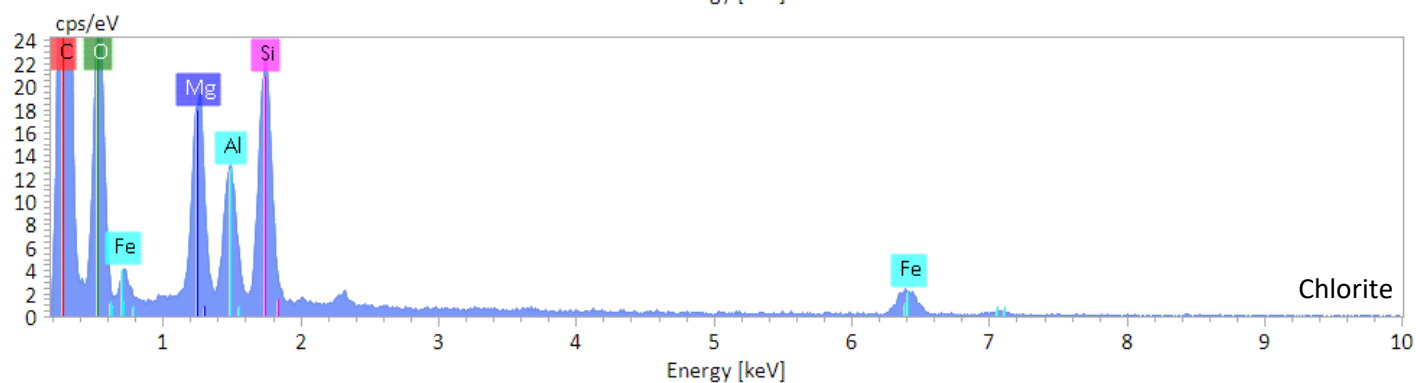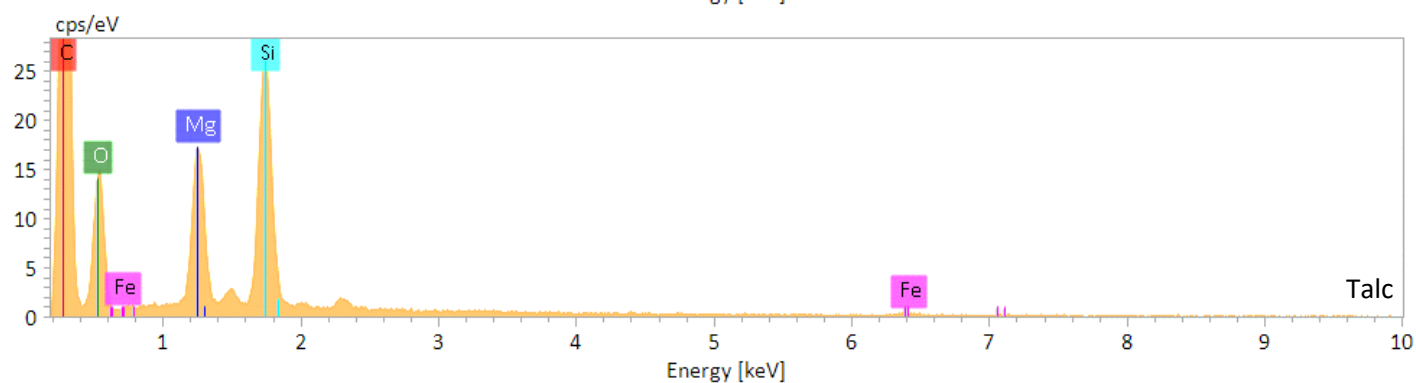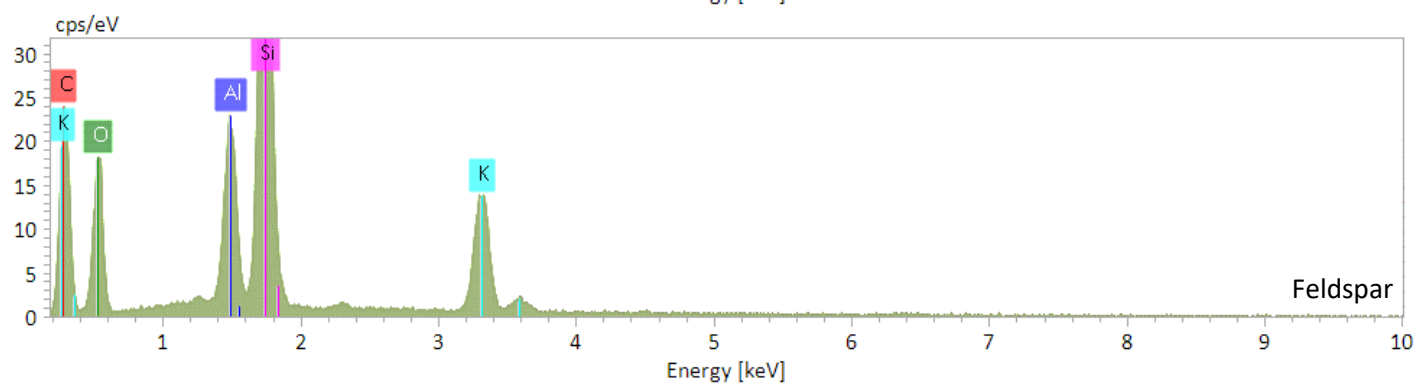

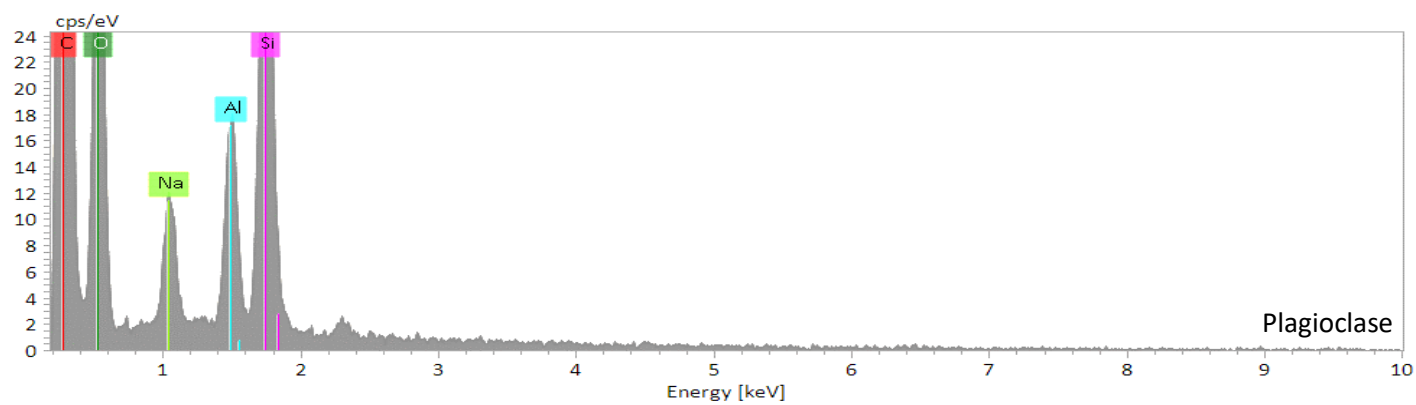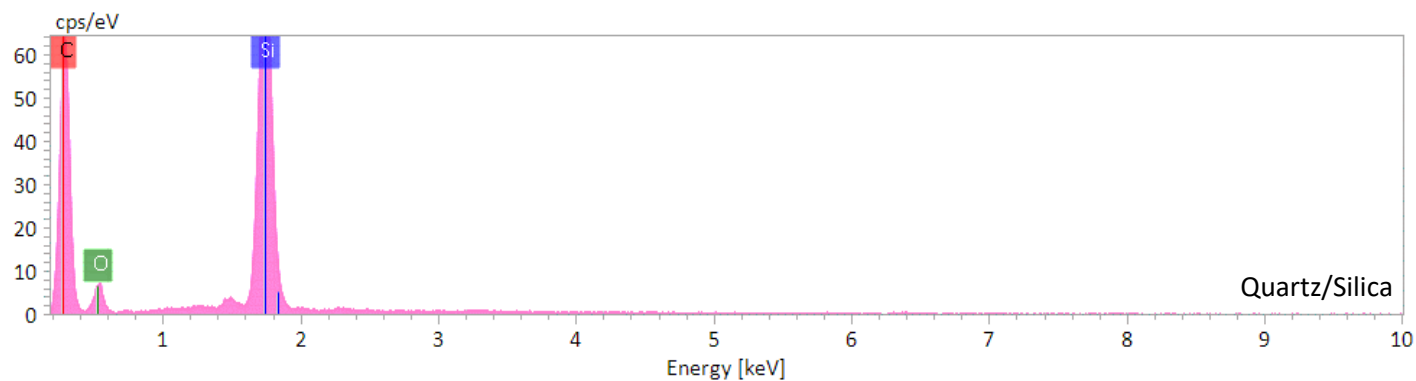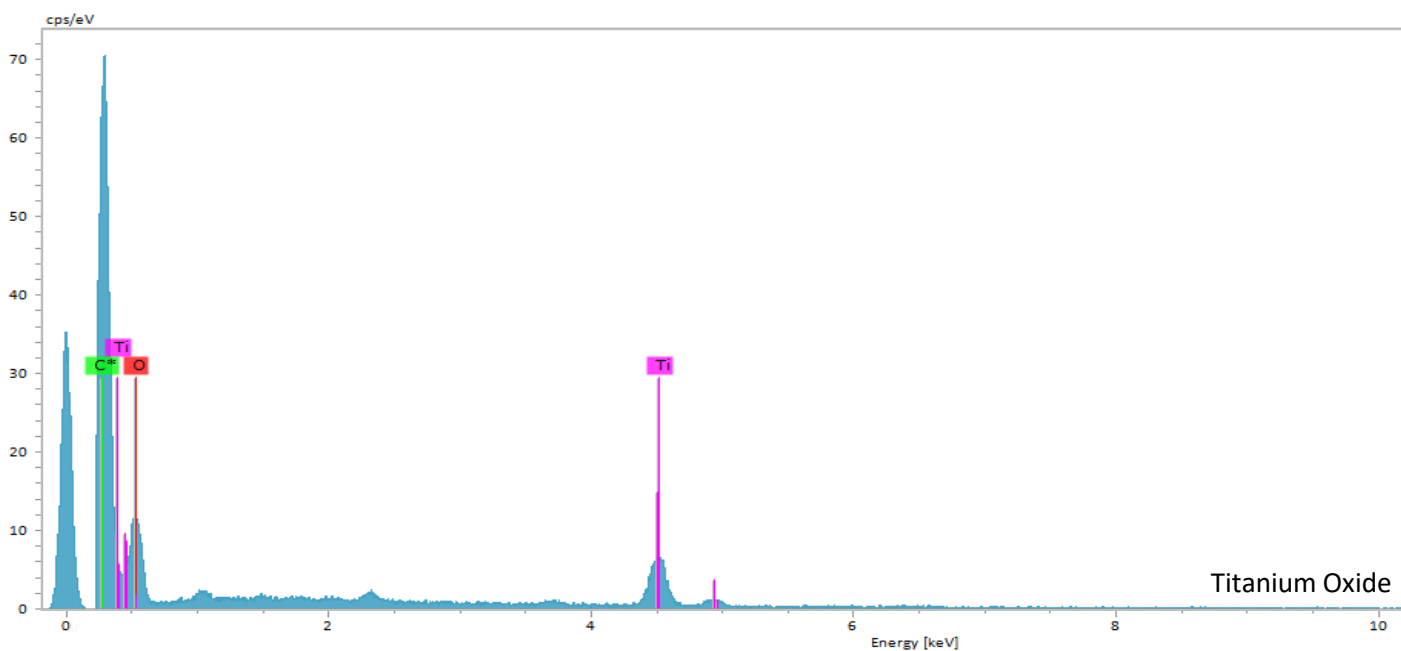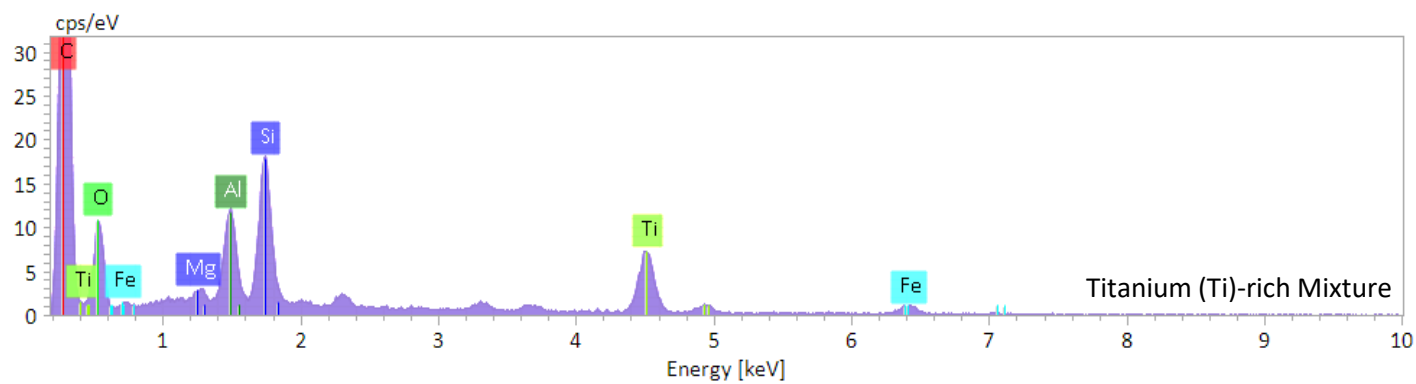

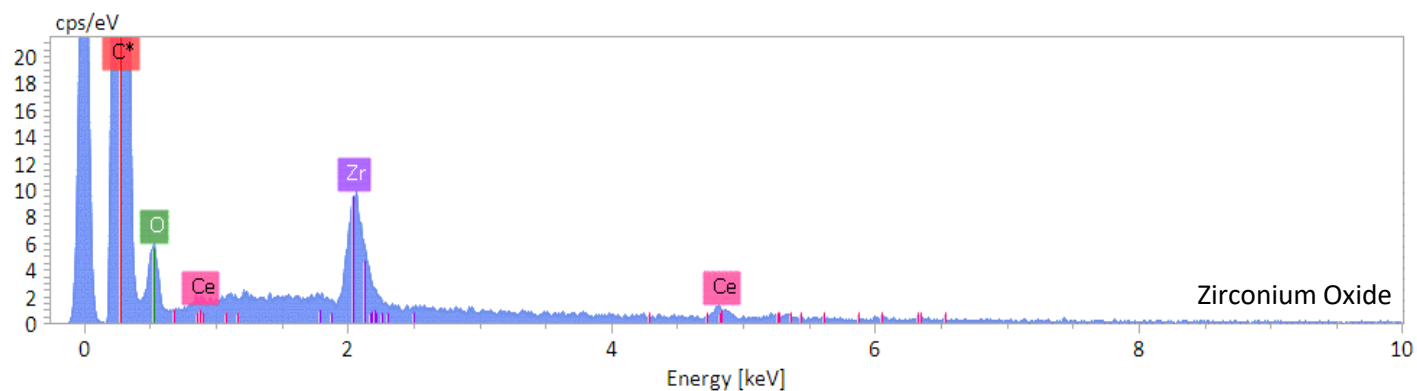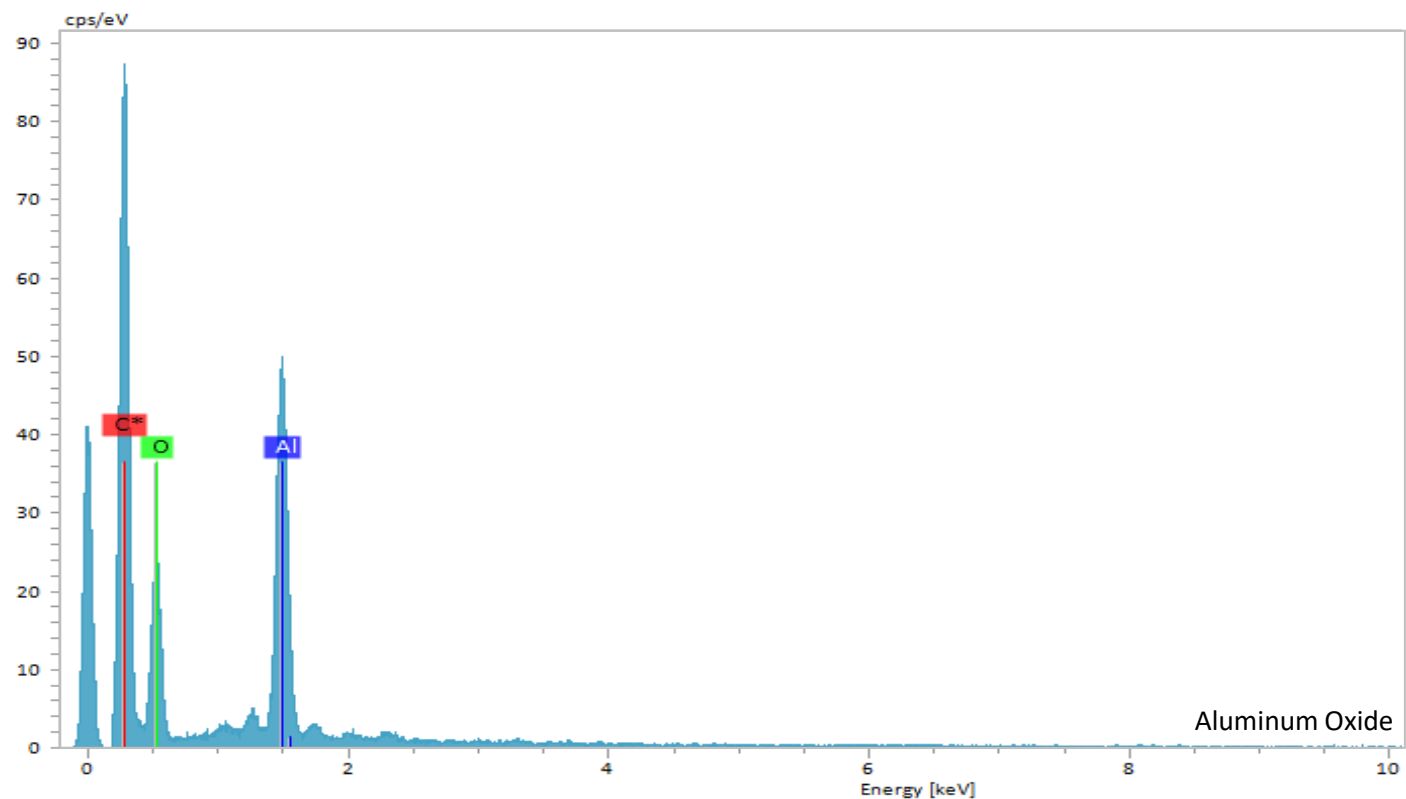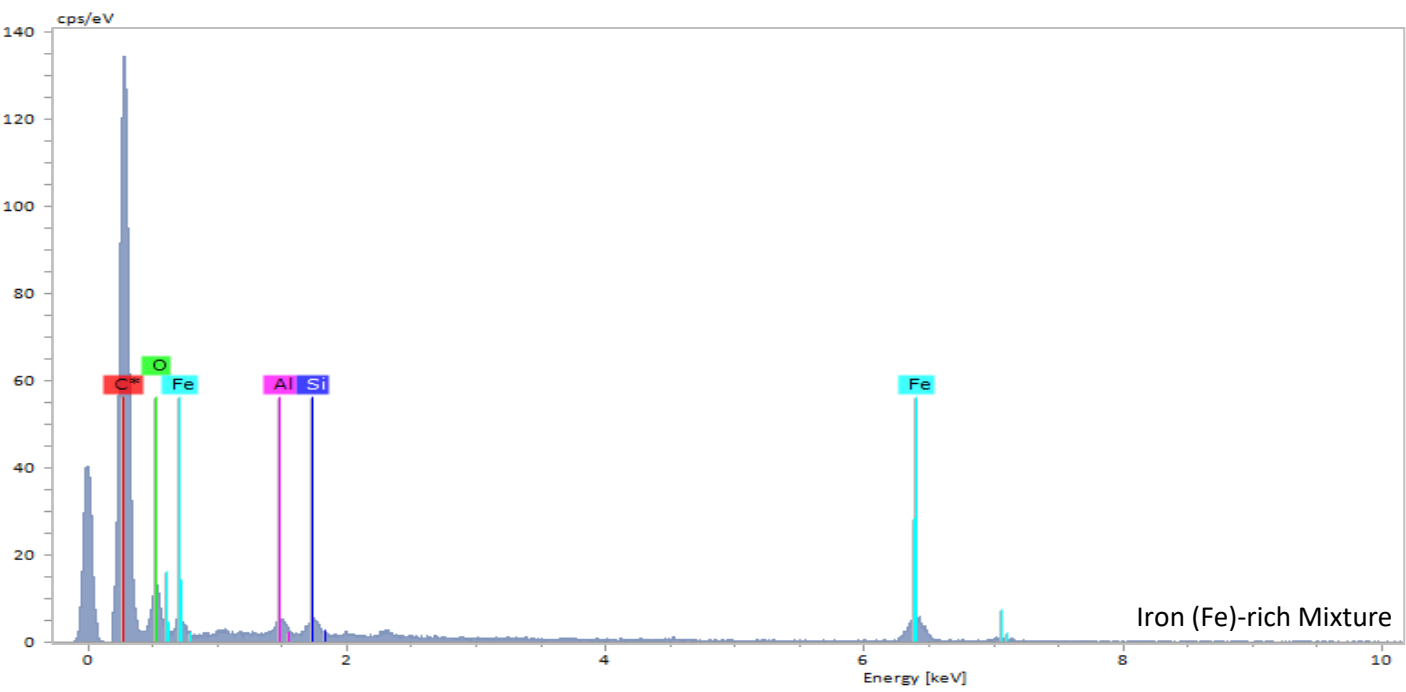

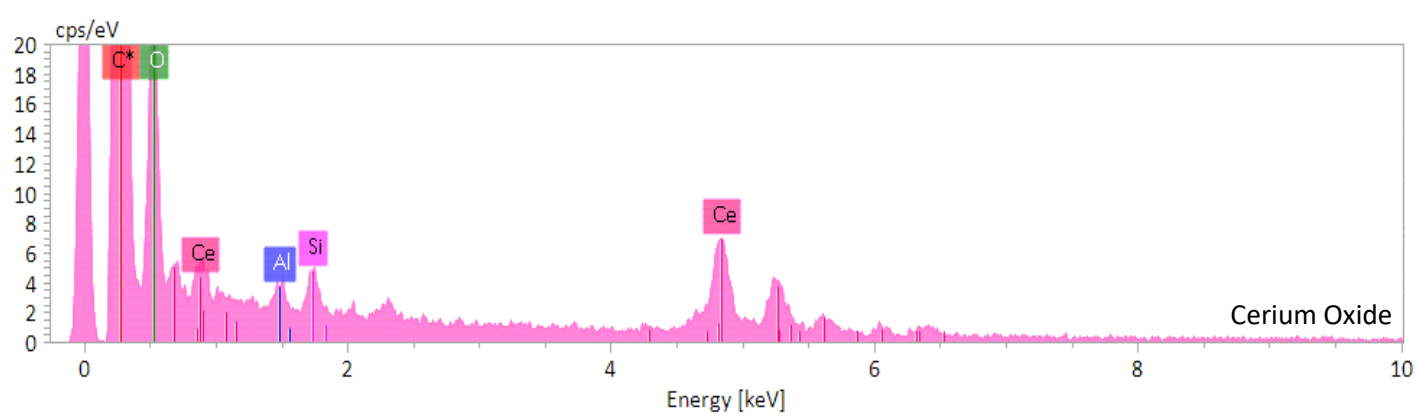

Supplement: S2 File — (PDF) [file pone.0301868.s011.pdf]
