# Supplementary material for: Particle analysis of surgical lung biopsies from deployed and non-deployed US service members during the Global War on Terrorism
Source: PLoS One. 2024 Apr 11;19(4):e0301868. doi: 10.1371/journal.pone.0301868 (PMC11008878; doi:10.1371/journal.pone.0301868)
Supplement: S1 Table — Estimates are from separate multivariable linear mixed effects models of the log-transformed outcome as the dependent variable and all characteristics as independent variables with a random intercept for service member. SE = standard error. Other service branches = Navy, Marine Corps, or Air Force. (DOCX) [file pone.0301868.s009.docx]

S1 Table. Estimated associations of service member and biopsy site characteristics with total and type-specific particle counts.

| **Outcome** | **Beta** | **SE** | **p** |
| --- | --- | --- | --- |
| ***Total*** |  |  |  |
| **DSM** | 0.90 | 0.37 | **0.02** |
| **Smoker** | 0.15 | 0.40 | 0.72 |
| **Army vs Others** | 0.64 | 0.41 | 0.12 |
| **Side – Right vs Left** | 0.58 | 0.46 | 0.21 |
| **Lobe – Middle vs Lower** | -0.61 | 0.45 | 0.19 |
| **Lobe – Upper vs Lower** | 0.29 | 0.33 | 0.37 |
| ***Illite*** |  |  |  |
| **DSM** | 1.58 | 0.54 | **<0.01** |
| **Smoker** | 0.16 | 0.58 | 0.78 |
| **Army vs Others** | 0.95 | 0.59 | 0.11 |
| **Side – Right vs Left** | 0.59 | 0.65 | 0.37 |
| **Lobe – Middle vs Lower** | -0.71 | 0.56 | 0.21 |
| **Lobe – Upper vs Lower** | -0.03 | 0.40 | 0.94 |
| ***Kaolinite*** |  |  |  |
| **DSM** | 0.85 | 0.37 | **0.03** |
| **Smoker** | 0.02 | 0.40 | 0.97 |
| **Army vs Others** | 0.80 | 0.41 | 0.06 |
| **Side – Right vs Left** | 0.45 | 0.46 | 0.34 |
| **Lobe – Middle vs Lower** | -0.82 | 0.48 | 0.09 |
| **Lobe – Upper vs Lower** | 0.27 | 0.34 | 0.44 |
| ***Iron (Fe)-rich mixture*** |  |  |  |
| **DSM** | 0.73 | 0.55 | 0.19 |
| **Smoker** | 0.17 | 0.59 | 0.78 |
| **Army vs Others** | 0.79 | 0.58 | 0.18 |
| **Side – Right vs Left** | 1.10 | 0.63 | 0.09 |
| **Lobe – Middle vs Lower** | -0.92 | 0.40 | **0.03** |
| **Lobe – Upper vs Lower** | 0.51 | 0.29 | 0.09 |
| ***Titanium Dioxide*** |  |  |  |
| **DSM** | 0.43 | 0.42 | 0.30 |
| **Smoker** | 0.09 | 0.45 | 0.85 |
| **Army vs Others** | 0.77 | 0.45 | 0.09 |
| **Side – Right vs Left** | 0.59 | 0.50 | 0.24 |
| **Lobe – Middle vs Lower** | -0.16 | 0.42 | 0.70 |
| **Lobe – Upper vs Lower** | 0.65 | 0.30 | **0.04** |
| ***Feldspar*** |  |  |  |
| **DSM** | 1.12 | 0.36 | **<0.01** |
| **Smoker** | 0.17 | 0.39 | 0.67 |
| **Army vs Others** | 0.34 | 0.39 | 0.39 |
| **Side – Right vs Left** | 0.49 | 0.44 | 0.27 |
| **Lobe – Middle vs Lower** | -0.67 | 0.43 | 0.12 |
| **Lobe – Upper vs Lower** | 0.16 | 0.31 | 0.61 |
| ***Quartz/Silica*** |  |  |  |
| **DSM** | 0.98 | 0.34 | **<0.01** |
| **Smoker** | -0.01 | 0.36 | 0.98 |
| **Army vs Others** | 0.90 | 0.37 | **0.02** |
| **Side – Right vs Left** | 0.46 | 0.41 | 0.26 |
| **Lobe – Middle vs Lower** | -0.55 | 0.38 | 0.15 |
| **Lobe – Upper vs Lower** | 0.43 | 0.27 | 0.12 |
| ***Titanium (Ti)-rich mixture*** |  |  |  |
| **DSM** | 1.61 | 0.47 | **<0.01** |
| **Smoker** | -0.42 | 0.51 | 0.41 |
| **Army vs Others** | -0.07 | 0.51 | 0.89 |
| **Side – Right vs Left** | 0.00 | 0.56 | 1.00 |
| **Lobe – Middle vs Lower** | -0.61 | 0.46 | 0.19 |
| **Lobe – Upper vs Lower** | 0.16 | 0.33 | 0.63 |

Estimates are from separate multivariable linear mixed effects models of the log-transformed outcome as the dependent variable and all characteristics as independent variables with a random intercept for service member. SE = standard error. Other service branches = Navy, Marine Corps, or Air Force.
